# Molecular design to regulate the photophysical properties of multifunctional TADF emitters towards high-performance TADF-based OLEDs with EQEs up to 22.4% and small efficiency roll-offs†
†Electronic supplementary information (ESI) available: experimental details including the synthetic procedure and DFT calculation, as well as device performance, thermal, photophysical, and electrochemical data. See DOI: 10.1039/c7sc04669c


**DOI:** 10.1039/c7sc04669c

**Published:** 2017-12-13

**Authors:** Ling Yu, Zhongbin Wu, Guohua Xie, Weixuan Zeng, Dongge Ma, Chuluo Yang

**Affiliations:** a Hubei Collaborative Innovation Center for Advanced Organic Chemical Materials , Hubei Key Lab on Organic and Polymeric Optoelectronic Materials , Department of Chemistry , Wuhan University , Wuhan , 430072 , People’s Republic of China . Email: clyang@whu.edu.cn; b State Key Laboratory of Polymer Physics and Chemistry , Changchun Institute of Applied Chemistry , University of Chinese Academy of Sciences , Changchun 130022 , People’s Republic of China . Email: mdg1014@ciac.ac.cn; c Institute of Polymer Optoelectronic Materials and Devices , State Key Laboratory of Luminescent Materials and Devices , South China University of Technology , Guangzhou 510640 , People’s Republic of China

## Abstract

Simultaneously achieving high efficiency and low efficiency roll-off remains a big challenge for OLEDs based on thermally activated delayed fluorescence (TADF) emitters. To address this issue, we elaborately designed and synthesized a series of new emitters with both TADF and aggregation-induced emission (AIE) properties by introducing 9,9-dimethyl-9,10-dihydroacridine (DMAC) or 10*H*-phenoxazine (PXZ) as donor units into a quinoxaline framework. By tuning the electron-donating capability of the donor as well as the amount of donor unit, the photophysical properties of the TADF-AIE emitters can be systematically regulated, with emissions ranging from green to red. We demonstrated efficient doped OLEDs with a maximum EQE of 22.4%, a maximum current efficiency (CE_max_) of 80.3 cd A^–1^ and a maximum power efficiency (PE_max_) of 64.1 lm W^–1^ for the green device, and an EQE_max_ of 14.1%, a CE_max_ of 36.1 cd A^–1^ and a PE_max_ of 28.1 lm W^–1^ for the orange device. Remarkably, these orange devices rendered small roll-offs of 1.4% and 21.3% respectively at a luminance of 100 and 1000 cd m^–2^. Attributed to the unique TADF and AIE features, the non-doped devices perform outstandingly with an EQE_max_ of 12.0%, a CE_max_ of 41.2 cd A^–1^ and a PE_max_ of 45.4 lm W^–1^.

## Abstract 2

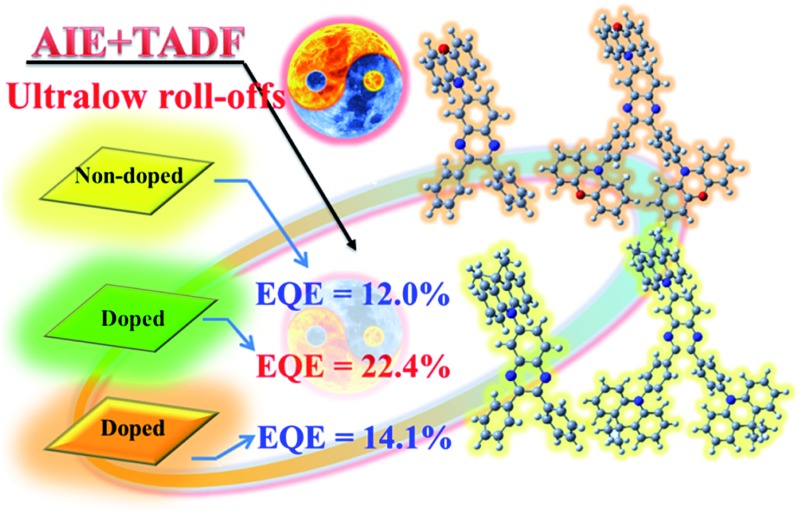
The photophysical properties of four new quinoxaline derivatives featuring both AIE and TADF characteristics were controlled to give high EQEs.

## Introduction

In the past few years, thermally activated delayed fluorescence (TADF) emitters have attracted intensive interest in the field of organic light-emitting diodes (OLEDs).^1^–^5^ Owing to the effective triplet exciton up-conversion process, they can achieve 100% internal quantum efficiency (IQE). The efficiencies of TADF-based fluorescent OLEDs have become comparable to those of OLEDs based on phosphorescent complexes.^6^–^9^ However, similar to phosphorescent OLEDs, TADF-based OLEDs also suffer from triplet-exciton-involved annihilation processes, which hamper the efficiency improvement and also inflict serious efficiency roll-off, especially for TADF-based orange/red OLEDs. Even though many efforts have been devoted, the current development of orange/red TADF emitters is far from satisfactory due to their low efficiency and serious efficiency roll-offs. As is known, it is inherently difficult for long-wavelength TADF emitters to simultaneously achieve a high fluorescence radiative rate (*k*_r_^S^) and a small singlet–triplet energy gap (Δ*E*_ST_). A restricted orbital overlap is in favor of a small Δ*E*_ST_, but it is not conducive to a high *k*_r_^S^. According to the energy-gap law,^10^ the non-radiative internal conversion rate (*k*_IC_) is significantly enhanced with an increase of emission wavelength, while *k*_r_^S^ is usually not high enough to overcome *k*_IC_ which results in a low photoluminescence (PL) quantum efficiency (*Φ*_PL_).^11^–^13^ Therefore, the most critical factor for the development of efficient red/orange TADF emitters is to simultaneously achieve a small Δ*E*_ST_ and a high fluorescence radiative rate *k*_r_^S^, which still requires unremitting endeavors.

To overcome the above-mentioned dilemma, the design of emitters integrating both TADF and aggregation-induced emission (AIE) features may be a wise approach, which not only can utilize 100% IQE, but can also effectively relieve the exciton quenching,^14^ especially at high brightness. Herein, we managed to respectively introduce 9,9-dimethyl-9,10-dihydroacridine (DMAC) and 10*H*-phenoxazine (PXZ) as donor units into a quinoxaline-based acceptor. As demonstrated, DMAC and PXZ have large spatial conformations,^15^,^16^ which are conducive to promote the separation of the highest occupied molecular orbitals (HOMOs) and the lowest unoccupied molecular orbitals (LUMOs) and thus achieve a small Δ*E*_ST_ in the donor–acceptor (D–A) system. We anticipate that the different electron-donating capabilities of DMAC and PXZ along with the amount of donor unit regulate the degree of intramolecular charge transfer (ICT) and consequently obtain orange/red TADF emitters with improved *Φ*_PL_. We also anticipate that the possible TADF-AIE emitters enable high-performance OLEDs with high efficiencies and low efficiency roll-offs.

## Results and discussion

### Synthesis and thermal properties

The four new compounds, namely 6-(9,9-dimethyl-9,10-dihydroacridinyl-10-yl)-2,3-diphenylquinoxaline (SBDBQ-DMAC), 2,3-bis(4-(9,9-dimethyl-9,10-dihydroacridinyl-10-yl)phenyl)-6-(9,9-dimethyl-9,10-dihydrogen-acridine-10-yl)-quinoxaline (DBQ-3DMAC), 6-(10*H*-phenoxazin-10-yl)-2,3-diphenylquinoxaline (SBDBQ-PXZ), and 2,3-bis(4-(10*H*-phenoxazin-10-yl)phenyl)-6-(10*H*-phenoxazin-10-yl)-quinoxaline (DBQ-3PXZ), were elaborately designed and synthesized by nucleophilic substitution reactions of the key intermediates (SBDBQ and 3BrDBQ) with DMAC and PXZ units, respectively (Scheme S1, ESI†).^17^,^18^ Their chemical structures were identified by ^1^H NMR, ^13^C NMR, mass spectrometry and elemental analysis. The four compounds of SBDBQ-DMAC, DBQ-3DMAC, SBDBQ-PXZ and DBQ-3PXZ exhibited high thermal decomposition temperatures (*T*_d_s, corresponding to 5% weight loss), *i.e.*, 370 °C, 424 °C, 363 °C and 455 °C, respectively, which have a positive relationship with their molecular weights. Their glass transition temperatures (*T*_g_s) also directly exemplified the above tendency (Fig. S1, ESI†). For example, the mono-substituted SBDBQ-DMAC and SBDBQ-PXZ have *T*_g_s of 88 and 84 °C, respectively. In contrast, the multi-substituted DBQ-3DMAC and DBQ-3PXZ render drastically increased *T*_g_s which are as high as 161 and 162 °C, respectively, indicating that the multi-substituted quinoxaline derivatives have better thermal stability and will be favorable for obtaining more stable organic electroluminescent devices.

### DFT calculations and electrochemical properties

**Fig. 1:**
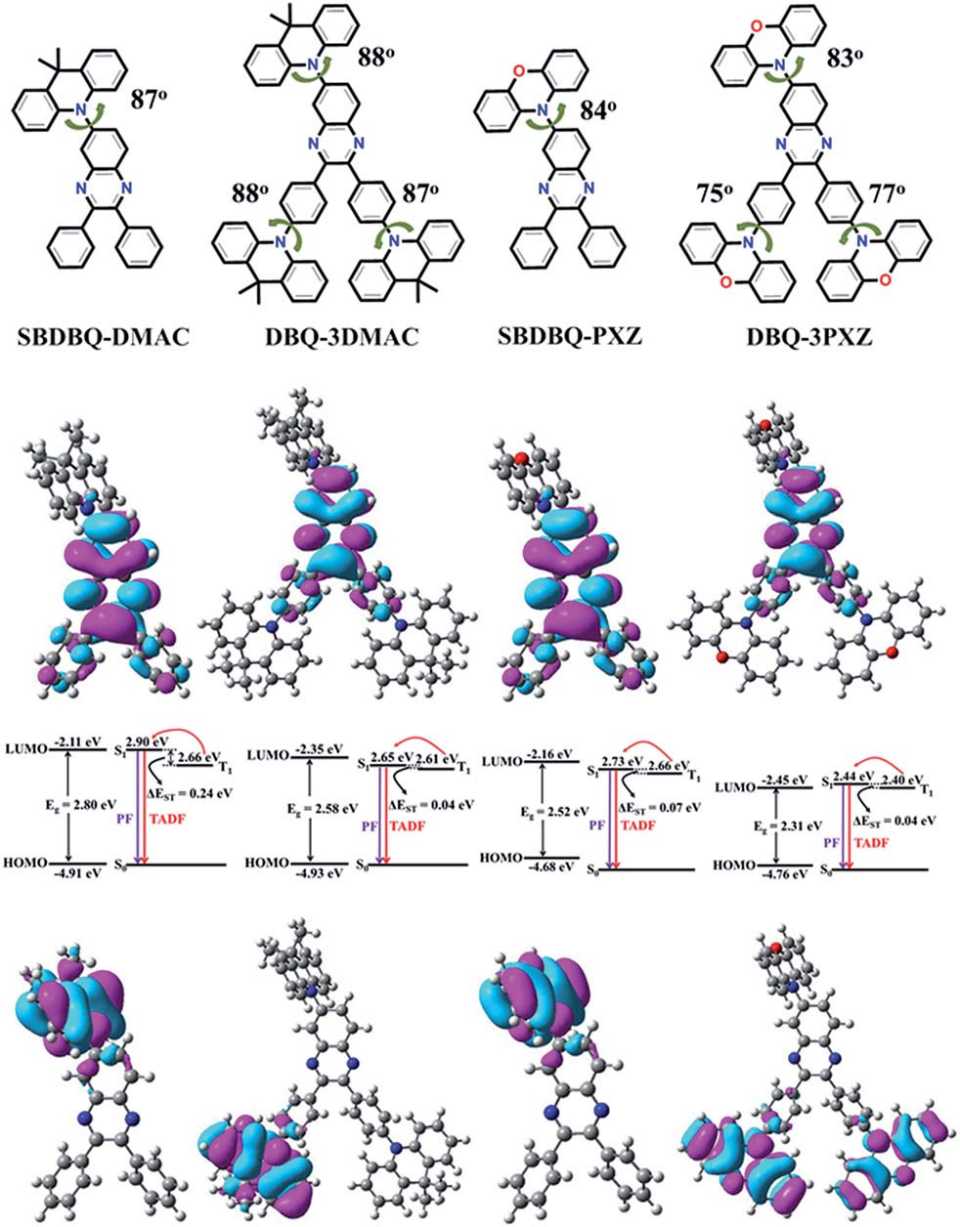
Molecular structures (upper), and the HOMO/LUMO distributions evaluated using the B3LYP/6-31g(d) level, and their energy level diagram for low-lying singlet and triplet excited states calculated with TD-B3LYP/6-31g(d).

In order to investigate the impacts of different electron-donating groups, the frontier molecular orbitals (FMOs) and energy levels of all the compounds were calculated using the B3LYP/6-31g(d) level. Their HOMOs and LUMOs are mainly distributed on the donor unit and the quinoxaline acceptor fraction, respectively. As is known, the value of Δ*E*_ST_ is proportional to the exchange interaction integral between the HOMO and the LUMO wavefunction in a molecule.^19^ As shown in Fig. 1, the four molecules display structures with large steric hindrance. For instance, the dihedral angles between the donor unit connected to the 6-position of quinoxaline and the central quinoxaline plane are 87°, 88°, 84° and 83° for SBDBQ-DMAC, DBQ-3DMAC, SBDBQ-PXZ and DBQ-3PXZ, respectively. The dihedral angles between the two benzene rings and the donor units are 88°/87° for DBQ-3DMAC, and 75°/77° for DBQ-3PXZ.^20^ Consistent with the very twisted structures, the theoretical Δ*E*_ST_s are about 0.24 eV for SBDBQ-DMAC, 0.04 eV for DBQ-3DMAC, 0.07 eV for SBDBQ-PXZ and 0.04 eV for DBQ-3PXZ (Table S1, ESI†). It is worth noting that DBQ-3DMAC and DBQ-3PXZ based on multi-donor substitution show a smaller Δ*E*_ST_ than their mono-donor substituted analogues.

The electrochemical properties of all the compounds were probed by cyclic voltammetry (Fig. S2†), and their HOMO levels were calculated from the oxidation peaks (Table S2, ESI†). Consistent with the theoretical analysis, their HOMO levels rise accordingly with the enhancement of electron-donating ability and the number of donor units with the order of –5.28 eV (SBDBQ-DMAC) < –5.26 eV (DBQ-3DMAC) < –5.17 eV (SBDBQ-PXZ) < –5.14 eV (DBQ-3PXZ).

### Photophysical and AIE properties

**Fig. 2:**
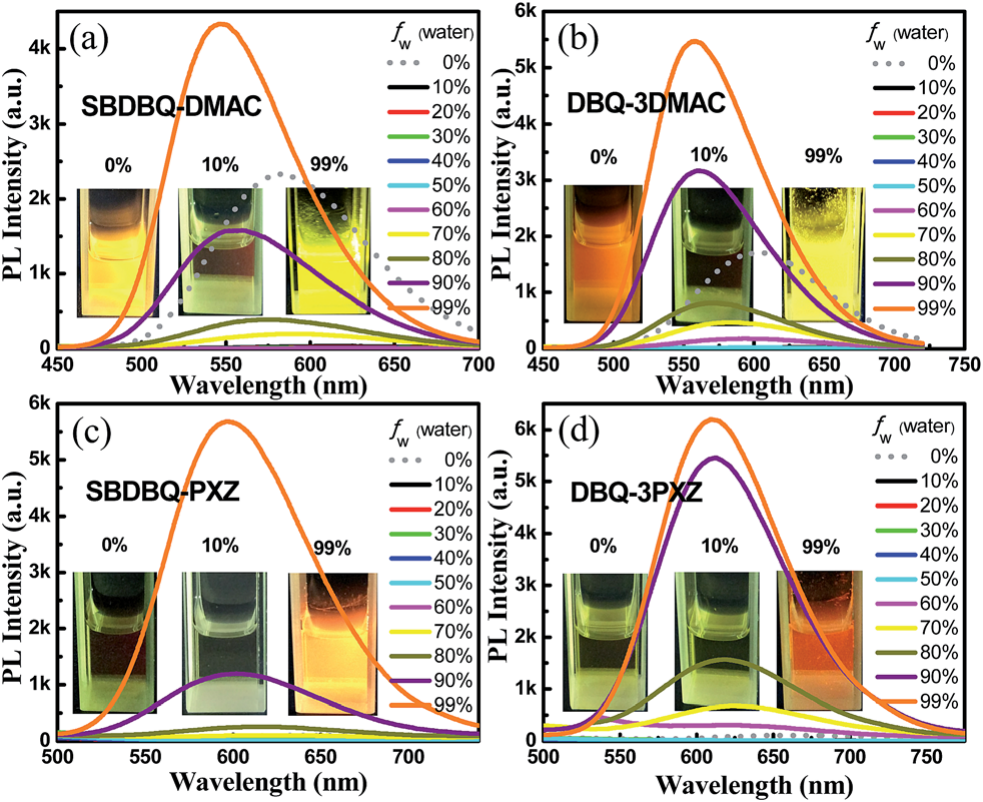
PL spectra of (a) SBDBQ-DMAC, (b) DBQ-3DMAC, (c) SBDBQ-PXZ and (d) DBQ-3PXZ in THF/water mixtures with different water fractions (*f*_w_).

The UV-vis absorption spectra, fluorescence and phosphorescence spectra of the four compounds in film are shown in Fig. S3 and S4.† Their strong absorption peaks at about 340 nm are attributed to the π–π* transition of the donor unit, and the weak absorption around the 400–550 nm range is due to the intramolecular charge transfer (ICT) process from the electron donor unit to the acceptor center. As the electron-donating ability increases, the spectra gradually exhibit bathochromic shift. In film, the fluorescence emission peaks of SBDBQ-DMAC, DBQ-3DMAC, SBDBQ-PXZ and DBQ-3PXZ are 541, 551, 594 and 618 nm, respectively, exhibiting a wide range of color tuning from green to yellow to orange to red. The phosphorescence spectra at 77 K of all the compounds are structureless, illustrating that the phosphorescence derives from the charge transfer (CT) state radiation transition of the triplet excitons. According to their fluorescence and phosphorescence spectra, the Δ*E*_ST_s of SBDBQ-DMAC, DBQ-3DMAC, SBDBQ-PXZ and DBQ-3PXZ are 0.06, 0.06, 0.07, and 0.03 eV, respectively, which are basically consistent with the results of the theoretical calculations. The thermal, photophysical and electrochemical data related to the compounds are summarized in Table S2, (ESI†). To probe the possible AIE phenomenon, we measured the PL spectra of these compounds in THF/water with various water fractions from 0 to 99%. As shown in Fig. 2, the PL intensities are abruptly enhanced as the water ratio reaches 90%, indicating a prominent AIE feature.

### TADF characterization

**Fig. 3:**
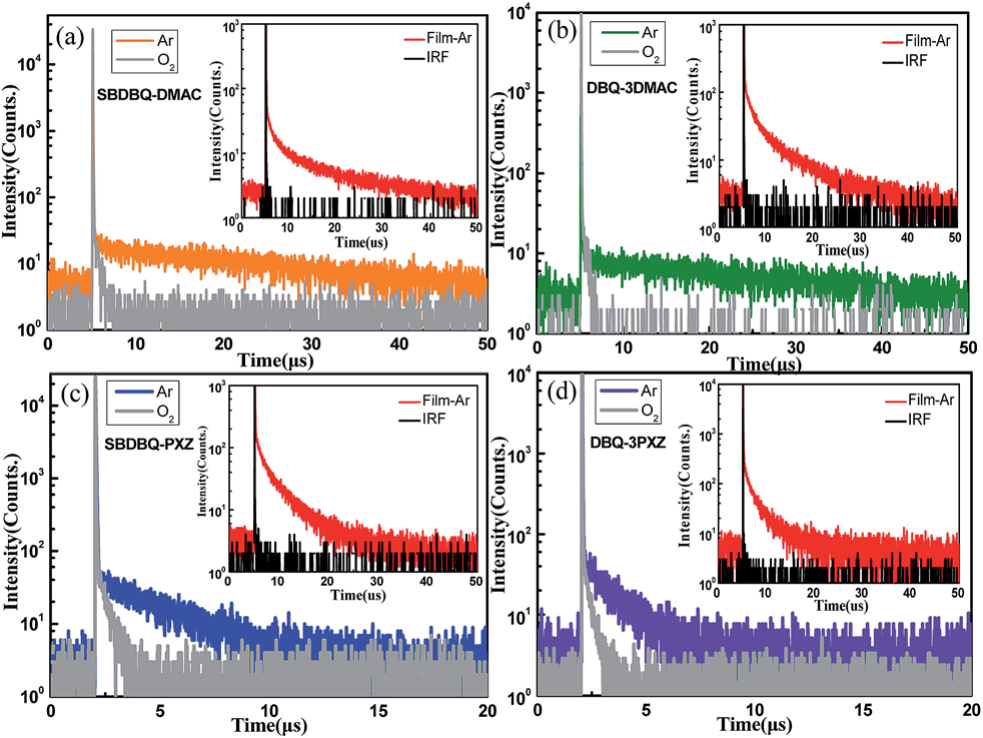
Transient PL characteristics of (a) SBDBQ-DMAC, (b) DBQ-3DMAC, (c) SBDBQ-PXZ, and (d) DBQ-3PXZ in toluene (10^–5^ M) under oxygen and argon at room temperature. The insets show the transient PL decay of the corresponding compound in film.

**Fig. 4:**
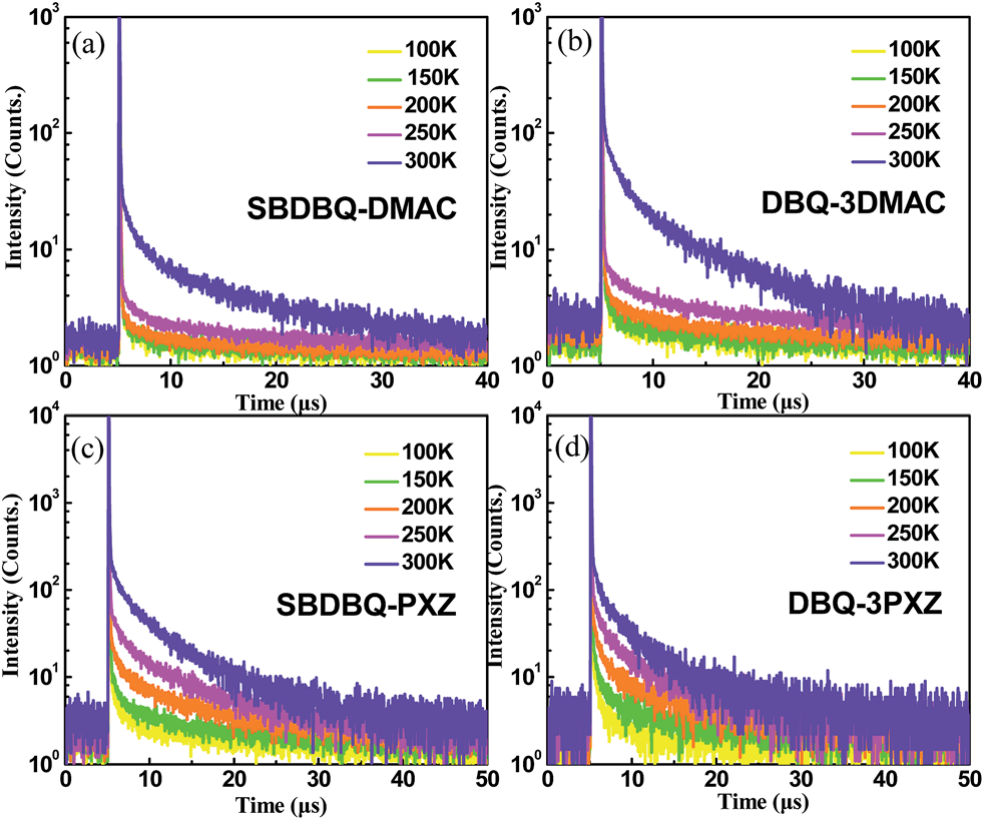
Temperature-dependent transient PL decays of (a) SBDBQ-DMAC, (b) DBQ-3DMAC, (c) SBDBQ-PXZ, and (d) DBQ-3PXZ from 100 to 300 K in film.

To verify their TADF characteristics, we tested the transient PL spectra in 10^–5^ M toluene solution. As exemplified in Fig. 3a–d, when oxygen is present, each of the four compounds only shows a prompt fluorescence emission.^21^ After bubbling with argon in toluene, all the compounds distinctly display prompt and delayed components with lifetimes of 23 ns/25.7 μs for SBDBQ-DMAC, 28 ns/33 μs for DBQ-3DMAC, 20 ns/2.86 μs for SBDBQ-PXZ and 8.7 ns/1.38 μs for DBQ-3PXZ. Obviously, the DMAC-based compounds exhibit much longer delayed fluorescence lifetimes than the PXZ-based compounds. In the neat film, their transient photoluminescence (PL) curves also exhibit double-exponential decay. Moreover, the delayed components are gradually intensified with the increase of ambient temperature from 100 to 300 K (Fig. 4), demonstrating the typical thermally activated nature.^1^,^22^

To further evaluate the TADF performances of the four compounds as emitters in the host–guest system, we also investigated the transient PL decays of the 10% emitters doped into CBP (4,4′-*N*,*N*′-dicarbazole-biphenyl), which consist of a nanosecond-scale prompt component and a microsecond-scale delayed component (Fig. S5†). Similar to the case in toluene, the delayed fluorescence lifetimes of the DMAC-based emitters are much longer than those of the PXZ-based emitters in the doped film. The *Φ*_PL_s of SBDBQ-DMAC, DBQ-3DMAC, SBDBQ-PXZ and DBQ-3PXZ in the doped films are 74%, 84%, 73% and 76%, respectively. As shown in Table 1, the radiative decay rate constants (*k*_r_^S^s) from the S_1_ to S_0_ transition and the rate constants (*k*_RISC_s) for RISC between the S_1_ and T_1_ states can be reasonably estimated as 2.1 × 10^7^/1.3 × 10^5^ s^–1^ (SBDBQ-DMAC), 2.0 × 10^7^/1.9 × 10^5^ s^–1^ (DBQ-3DMAC), 9.7 × 10^6^/8.4 × 10^5^ s^–1^ (SBDBQ-PXZ) and 1.1 × 10^7^/1.2 × 10^6^ s^–1^ (DBQ-3PXZ).^23^ These above-mentioned experimental results reveal that DBQ-3DMAC possesses the highest *Φ*_PL_ and DBQ-3PXZ features the maximum *k*_RISC_, which suggest that they could achieve better electroluminescent performance.

**Table 1:** The lifetimes, quantum efficiencies and rate constants of CBP: 10% TADF in film

TADF compounds	*τ*_F_ [ns]	*τ*_d_ [μs]	*Φ*_PL_ ^*a*^ [%]	*Φ*_DF_ ^*a*^ [%]	*k*_P_ ^*b*^ [s^–1^]	*k*_d_ ^*b*^ [s^–1^]	*k*_r_^S^ ^*c*^ [s^–1^]	*k*_ISC_ ^*d*^ [s^–1^]	*k*_RISC_ ^*d*^ [s^–1^]
SBDBQ-DMAC	23	8.3	74	26	4.3 × 10^7^	1.2 × 10^5^	2.1 × 10^7^	2.2 × 10^7^	1.3 × 10^5^
DBQ-3DMAC	26	6.5	84	32	3.8 × 10^7^	1.5 × 10^5^	2.0 × 10^7^	1.8 × 10^7^	1.9 × 10^5^
SBDBQ-PXZ	32	2.4	73	42	3.1 × 10^7^	4.2 × 10^5^	9.7 × 10^6^	2.1 × 10^7^	8.4 × 10^5^
DBQ-3PXZ	29	1.9	76	46	3.4 × 10^7^	5.3 × 10^5^	1.0 × 10^7^	2.4 × 10^7^	1.2 × 10^6^

^*a*^The total and delayed fluorescence quantum yield, respectively.

^*b*^The rate constant for prompt and delayed fluorescence, respectively.

^*c*^ *k*_r_^S^ represents the radiative decay rate constant from S_1_ to S_0_ transition.

^*d*^The rate constants for intersystem crossing (ISC) and reverse intersystem crossing (RISC) between the S_1_ and T_1_ states, respectively.

### Device characterization

**Fig. 5:**
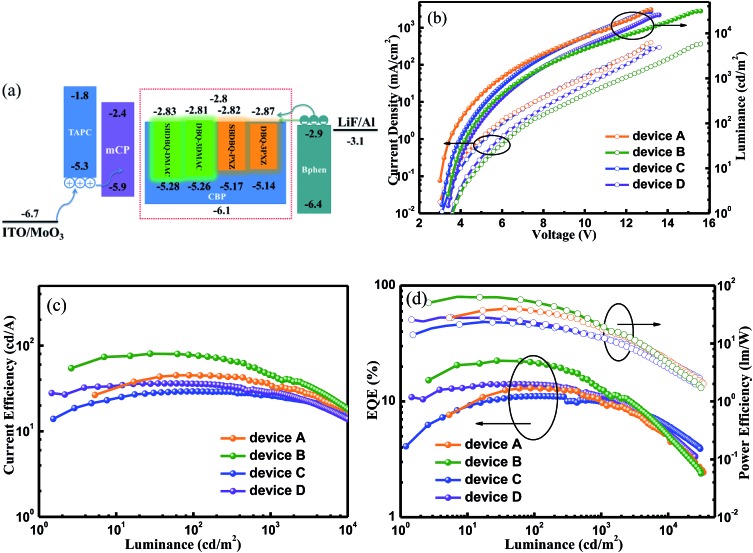
(a) A schematic diagram of the energy levels of the doped devices (A–D), (b) current density–voltage–brightness characteristics, (c) current efficiency *versus* luminance curves, and (d) EQE and power efficiency *versus* luminance curves.

The satisfactory *Φ*_PL_s and rate constants inspired us to investigate the potential applications in doped OLEDs (device A for SBDBQ-DMAC, B for DBQ-3DMAC, C for SBDBQ-PXZ and D for DBQ-3PXZ, Fig. 5a) with the following configuration: ITO/MoO_3_ (10 nm)/TAPC (50 nm)/mCP (10 nm)/CBP: TADF emitter (10%, 20 nm)/Bphen (45 nm)/LiF (1 nm)/Al, where TAPC (1,1-bis[4-[*N*,*N*-di(*p*-tolyl)-amino]phenyl]cyclohexane) and Bphen (4,7-diphenyl-1,10-phenanthroline) were used as hole- and electron-transport materials, respectively, and mCP (1,3-bis(*N*-carbazoly)benzene) was used as an electron/exciton blocking layer. Fig. 5b shows the current density–voltage–brightness curves of the doped devices A–D. Under electrical excitation, the turn-on voltage and maximum brightness of devices A–D are 3.0 V/33 586 cd m^–2^, 3.4 V/31 099 cd m^–2^, 3.1 V/30 039 cd m^–2^ and 3.4 V/25 375 cd m^–2^, respectively. All the devices exhibit excellent electroluminescent performance. The devices with SBDBQ-DMAC (device A) and DBQ-3DMAC (device B) present green light emission peaking at 532 and 536 nm, respectively. Meanwhile, the devices with SBDBQ-PXZ (device C) and DBQ-3PXZ (device D) show orange light emission with identical peaks at 572 nm (Fig. S6†). For the green TADF devices, the device A based on DBQ-DMAC achieves an EQE_max_, a CE_max_ and a PE_max_ of 13.0%, 45.0 cd A^–1^ and 39.9 lm W^–1^, respectively. The device B based on DBQ-3DMAC exhibits the highest EL performance with an EQE_max_ of 22.4%, a CE_max_ of 80.3 cd A^–1^ and a PE_max_ of 64.1 lm W^–1^ without any light out-coupling technique, which is significantly superior to device A, owing to the relatively high *Φ*_PL_ (84%) and *k*_RISC_ (1.9 × 10^5^ s^–1^). It is worth noting that both devices A and B display comparatively low efficiency roll-offs (Table 2).^24^ Furthermore, the orange devices C and D based on SBDBQ-PXZ and DBQ-3PXZ not only exhibit considerably high efficiencies, but also achieve ultralow efficiency roll-offs compared to any other reported orange TADF devices. As shown in Fig. 5 and Table 2, the device C achieves an EQE_max_ of 11.1%, a CE_max_ of 29.1 cd A^–1^ and a PE_max_ of 23.4 lm W^–1^, and the efficiencies maintain at 11.0%/10.1%, 28.8/26.6 cd A^–1^ and 20.8/12.9 lm W^–1^, at a brightness of 100 and 1000 cd m^–2^, respectively. This corresponds to the ultra-low efficiency roll-offs of 0.9% and 9.0%, respectively. Comparatively, the DBQ-3PXZ-based device D obtains higher efficiencies with an EQE_max_ of 14.1%, a CE_max_ of 36.1 cd A^–1^ and a PE_max_ of 28.1 lm W^–1^. Inspiringly, when the brightness is 100 and 1000 cd m^–2^, the EQE, CE and PE are still as high as 13.9%/11.1%, 35.3/28.4 cd A^–1^ and 22.9/12.4 lm W^–1^ with efficiency roll-offs of only 1.4% and 21.3%, respectively. The AIE nature and TADF property endows these target compounds with excellent solid-state PL efficiency and a high utilization of excitons under electrical excitation, meanwhile the relatively low efficiency roll-off probably results from the favourable charge injection, transport, and recombination ability at the high brightness. To the best of our knowledge, the excellent device performance and extremely low efficiency roll-offs are among the highest values for TADF-based OLEDs ever reported, especially for orange TADF devices (Table S3, ESI†). In terms of the relationship between the molecular structures and device performance, we can draw conclusions as below: (i) as predicted by theory, the multi-substituted quinoxaline derivatives, namely DBQ-3DMAC and DBQ-3PXZ, display better device efficiencies, which are related to their own *Φ*_PL_, *k*_r_^S^ and *k*_RISC_; (ii) the efficiency roll-offs of the devices with PXZ as the donor unit are smaller than those of the device with DMAC as the donor unit, attributed to the shorter lifetime, which is beneficial to suppress triplet-exciton-involved quenching, such as singlet–triplet annihilation (STA) and triplet–triplet annihilation (TTA) processes, and in a certain extent to alleviate the efficiency roll-offs.^25^

**Table 2:** The characteristic data of the doped devices (A–D)

Device	*V*_on_ ^*a*^ [V]	LE_max_ [cd m^–2^]	EQE ^*b*^ [%]	CE ^*b*^ [cd A^–1^]	PE ^*b*^ [lm W^–1^]	Peak [nm]	CIE [*x*, *y*]
A	3.0	33 586	13.0/12.9/10.0	45.0/44.9/34.6	39.9/35.9/18.7	532	(0.34, 0.60)
B	3.4	31 099	22.4/21.6/12.5	80.3/77.9/44.8	64.1/53.3/20.4	536	(0.35, 0.59)
C	3.1	30 039	11.1/11.0/10.1	29.1/28.8/26.6	23.4/20.8/12.9	572	(0.49, 0.50)
D	3.4	25 375	14.1/13.9/11.1	36.1/35.3/28.4	28.1/22.9/12.4	572	(0.50, 0.49)

^*a*^At a luminance of 1 cd m^–2^.

^*b*^The maximum value and the corresponding values at a brightness of 100 and 1000 cd m^–2^, respectively.

**Fig. 6:**
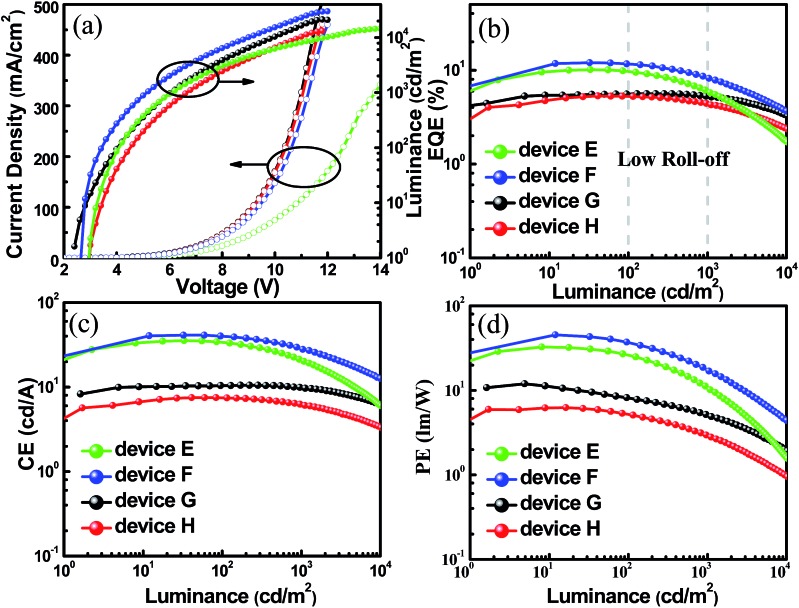
(a) Current density–voltage–brightness characteristics, (b) EQE *versus* luminance curves, (c) current efficiency *versus* luminance curves, and (d) power efficiency *versus* luminance curves of the non-doped devices (E–H).

In addition, the unique TADF-AIE features enlightened us to further explore non-doped devices, and a common structure was fabricated with the configurations of ITO/MoO_3_ (10 nm)/TAPC (50 nm)/mCP (10 nm)/TADF emitter (20 nm)/Bphen (45 nm)/LiF/Al (SBDBQ-DMAC for device E, DBQ-3DMAC for device F, SBDBQ-PXZ for device G and DBQ-3PXZ for device H). As shown in Fig. S7,† both devices E and F display yellow emission peaking at 544 and 548 nm, respectively. In contrast, devices G and H exhibit orange (peaking at 608 nm) and red (peaking at 616 nm) emission, respectively, which are consistent with their PL spectra in the neat films. Eminently, the best results are obtained by device F based on the multi-substituted DBQ-3DMAC, featuring an EQE_max_ of 12.0%, a CE_max_ of 41.2 cd A^–1^ and a PE_max_ of 45.4 lm W^–1^. Besides, device E based on the mono-substituted SBDBQ-DMAC achieves high performance with an EQE_max_ of 10.1%, a CE_max_ of 35.4 cd A^–1^ and a PE_max_ of 32.7 lm W^–1^ (Fig. 6 and Table 3). To the best of our knowledge, these efficiencies are among the highest reported for non-doped yellow OLEDs (Table S4, ESI†). As expected, the long-wavelength emissive devices G and H achieve an EQE_max_ of 5.6% and 5.3%, respectively, which all exceed the theoretical limit of 5% for traditional fluorescent emitters. Most importantly, all the non-doped devices exhibit low efficiency roll-offs, which may be attributed to their unique TADF-AIE nature.

**Table 3:** The characteristic data of the non-doped devices (E–H)

Device	*V*_on_ ^*a*^ [V]	LE_max_ [cd m^–2^]	EQE ^*b*^ [%]	CE ^*b*^ [cd A^–1^]	PE ^*b*^ [lm W^–1^]	Peak [nm]	CIE [*x*, *y*]
E	2.8	14 578	10.1/9.7/6.0	35.4/33.4/21.2	32.7/26.2/10.7	544	(0.39, 0.58)
F	2.6	29 843	12.0/11.9/8.3	41.2/40.4/28.5	45.4/36.7/17.6	548	(0.40, 0.57)
G	2.4	21 050	5.6/5.6/5.2	10.5/10.5/9.8	12.0/8.2/5.2	608	(0.56, 0.43)
H	2.8	13 167	5.3/5.2/4.4	7.5/7.4/6.2	6.2/5.3/2.9	616	(0.60, 0.40)

^*a*^At a luminance of 1 cd m^–2^.

^*b*^The maximum value and the corresponding values at a brightness of 100 and 1000 cd m^–2^, respectively.

## Conclusion

In conclusion, we developed a series of new asymmetric quinoxaline derivatives with donor units of DMAC and PXZ. Through fine tuning, we have successfully solved the contradiction between Δ*E*_ST_ and *k*_r_^S^ for TADF molecules featuring minimal Δ*E*_ST_s and high *Φ*_PL_s. The delayed fluorescence lifetimes of the TADF molecules employing PXZ as the electron donor are much shorter than those of the molecules with DMAC as the donor unit, either in solution or in film. The DBQ-3DMAC-based doped device exhibits the highest EQE of 22.4%. Moreover, the DBQ-3PXZ-based orange doped device shows an EQE_max_ of 14.1%, accompanied by extremely low efficiency roll-offs. To the best of our knowledge, this excellent performance is among the highest values for TADF-based devices ever reported, especially for red/orange TADF devices. Attributed to their simultaneous TADF and AIE features, the non-doped devices render excellent performance with an EQE_max_ of 12.0%, a CE_max_ of 41.2 cd A^–1^ and a PE_max_ of 45.4 lm W^–1^.

## Conflicts of interest

The authors declare no competing financial interest.

## Supplementary Material

Supplementary information
