# Supplementary material for: Molecular design to regulate the photophysical properties of multifunctional TADF emitters towards high-performance TADF-based OLEDs with EQEs up to 22.4% and small efficiency roll-offs
Source: Chem Sci. 2017 Dec 13;9(5):1385–91. doi: 10.1039/c7sc04669c (PMC5885939; doi:10.1039/c7sc04669c)
Supplement: Supplementary file 1 [file SC-009-C7SC04669C-s001.pdf]

Electronic Supplementary Material (ESI) for Chemical Science.
This journal is © The Royal Society of Chemistry 2018

Electronic Supplementary Information (ESI)

Molecular design to regulate the photophysical properties of multifunctional TADF emitters towards high-performance TADF-based OLEDs with EQEs up to 22.4% and small efficiency roll-offs

Ling Yu, Zhongbin Wu, Guohua Xie, Weixuan Zeng, Dongge Ma and Chuluo Yang**

Corresponding Author

**E-mail: clyang@whu.edu.cn (Chuluo Yang)*

**E-mail: mdg1014@ciac.ac.cn (Dongge Ma)*

General Information

The ^{1}H NMR and ^{13}C NMR spectra were recorded on a Bruker Advance Ⅲ (400 MHz) spectrometer with $CDCl_3$ as the solvent. Mass spectra were measured on a ZAB 3F-HF mass spectrophotometer. Matrix-assisted laser desorption ionization/time-of-flight (MALDI-TOF) mass spectra were performed on a Bruker BIFLEX III TOF mass spectrometer. Elemental analyses were performed on a Vario EL-III microanalyzer. Thermal gravity analysis (TGA) was performed on a Netzsch STA 449C instrument. Differential scanning calorimetry (DSC) was performed on a NETZSCH DSC 200 PC unit at a heating rate of 20 °C min^{-1} from 25 to 350 °C under argon. The glass transition temperature (T_g) was determined from the second heating scan at a heating rate of 10 °C min^{-1}. UV-vis absorption spectra were recorded on a Shimadzu UV-2700 recording spectrophotometer. Photoluminescence (PL) spectra were recorded on a Hitachi F-4600 fluorescence spectrophotometer. Cyclic voltammetry (CV) was carried out in nitrogen-purged dichloromethane (oxidation scan) at room temperature with a CHI voltammetric analyzer. N-Bu_4PF_6 (0.1 M) was used as the supporting electrolyte. The conventional three-electrode configuration consists of a platinum working electrode, a platinum wire auxiliary electrode, and an Ag wire pseudoreference electrode with ferrocenium-ferrocene (Fc^+/Fc) as the internal standard. The PL lifetimes were measured by a single photon counting spectrometer from Edinburgh Instruments (FLS920) with a Picosecond Pulsed UV-LASTER (LASTER377) as the excitation source. The photoluminescence quantum efficiency was measured using an absolute photoluminescence quantum yield measurement system (C9920-02, Hamamatsu Photonics).

Devices fabrication and characterization

The device was grown on clean glass substrates pre-coated with a 180-nm-thick ITO with a sheet resistance of 10 Ω per square. The ITO glass substrates were pre-cleaned carefully and treated by oxygen plasma for 2 min. Then the sample was transferred to the deposition system. For the doped devices, 10 nm MoO_3 was firstly deposited onto the ITO substrate, consecutively followed by TAPC (50 nm), mCP (10 nm), emissive layer (20 nm), and Bphen (45 nm). For the non-doped devices, 10 nm MoO_3 was firstly deposited onto the ITO substrate, consecutively followed by TAPC (50 nm), mCP (10 nm), emissive layer (20 nm), and Bphen (45 nm). Finally, a cathode composed of lithium fluoride and aluminum was sequentially deposited onto the sample in the vacuum of 10^{-6} Torr. The current-voltage-brightness characteristic was measured by using a Keithley source measurement unit (Keithley 2400 and Keithley 2000) with a calibrated silicon photodiode. The EL spectra were measured by a Spectrascan PR650 spectrophotometer. The EQE was calculated from the luminance, the EL spectrum, and the current density.

Synthesis of materials

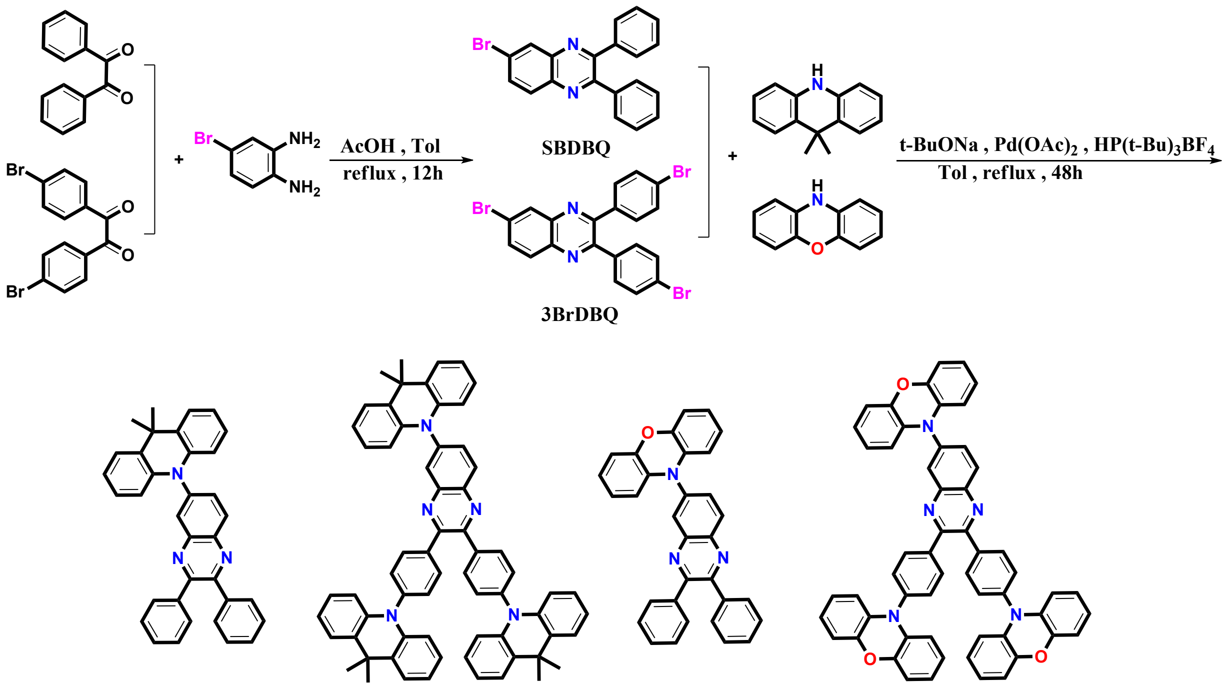

SBDBQ-DMAC **DBQ-3DMAC** **SBDBQ-PXZ** **DBQ-3PXZ**

Scheme S1. Synthetic routes of the target compounds.

6-(9,9-dimethyl-9,10-dihydroacridinyl-10-yl)-2,3-diphenylquinoxaline (**SBDBQ-DMAC**): A mixture of SBDBQ (1.30 g, 3.60 mmol), 9,9-dimethyl-9,10-dihydro-acridine (DMAC) (0.98 g, 4.67 mmol), $Pd(OAc)_2$ (20 mg, 0.09 mmol), HP(t-Bu)$_3$BF$_4$ (64 mg, 0.22 mmol), t-BuONa (0.52 g, 5.42 mmol), and toluene (150 mL) was refluxed under argon for 24 h. After cooled, the mixture was extracted with brine and CH_2Cl_2, and dried over anhydrous Na_2SO_4. After removal of the solvent, the residue was purified by column chromatography on silica gel using dichloromethane/petroleum ether (2:3 by vol.) as the eluent to give a yellow powder (1.70 g, yield: 97%). ^{1}H NMR (400 MHz, $CDCl_3$) δ [ppm]: 8.42 (d, J = 12.0 Hz, 1H), 8.23 (d, J = 4.0 Hz, 1H), 7.73-7.70 (m, 1H), 7.59-7.54 (m, 4H), 7.52-7.49 (m, 2H), 7.41-7.35 (m, 6H), 6.99-6.97 (m, 4H), 6.43-6.41 (m, 2H), 1.73 (s, 6H). ^{13}C NMR

(100 MHz, $CDCl_3$) δ [ppm]: 154.13, 153.90, 142.59, 142.51, 140.65, 138.91, 138.76, 129.91, 129.87, 128.44, 128.37, 126.46, 125.26, 121.17, 114.53, 36.15, 31.00, 30.90. MS (EI): *m/z* 489 [M^+]. Elemental analysis (%) for $C_{35}H_{27}N_3$: C 85.86, H 5.56, N 8.58; Found: C 85.99, H 5.57, N 8.51.

2,3-bis(4-(9,9-dimethyl-9,10-dihydroacridinyl-10-yl)phenyl)-6-(9,9-dimethyl-9,10-dihydrogen-acridine-10-yl)-quinoxaline (**DBQ-3DMAC**): it was prepared by the same procedure with **SBDBQ-DMAC** excepting using the key intermediate 3BrDBQ (1.00 g, 1.93 mmol) to replace SBDBQ. **DBQ-3DMAC** is a yellow powder (1.70 g, yield: 97%). ^{1}H NMR (400 MHz, $CDCl_3$) δ [ppm]: 8.51 (d, *J* = 8.0 Hz, 1H), 8.36 (d, *J* = 4.0 Hz, 1H), 7.91-7.84 (m, 5H), 7.54-7.52 (m, 2H), 7.47-7.41 (m, 8H), 7.05-6.99 (m, 4H), 6.92-6.82 (m, 8H), 6.51-6.49 (m, 2H), 6.34 (t, *J* = 8.0 Hz, 4H), 1.75 (s, 6H), 1.69 (d, *J* = 8.0 Hz, 12H). ^{13}C NMR (100 MHz, $CDCl_3$) δ [ppm]: 153.47, 153.36, 143.31, 142.71, 140.78, 138.61, 138.48, 132.53, 131.53, 131.44, 130.26, 126.56, 125.33, 125.15, 121.41, 120.86, 114.79, 114.04, 36.23, 35.95, 31.01, 30.92, 30.88, 30.82. MS (EI): *m/z* 905 [M^+]. Elemental analysis (%) for $C_{65}H_{33}N_5$: C 86.35, H 5.91, N 7.75; Found: C 86.75, H 6.05, N 7.85.

6-(10*H*-phenoxazin-10-yl)-2,3-diphenylquinoxaline (**SBDBQ-PXZ**): it was prepared by the same procedure with **SBDBQ-DMAC** excepting using 10*H*-phenoxazine (PXZ) (0.79 g, 4.31 mmol) to replace DMAC. **SBDBQ-PXZ** is an orange powder (1.46 g, yield: 95%). ^{1}H NMR (400 MHz, $CDCl_3$) δ [ppm]: 8.39 (d, *J* = 8.0 Hz, 1H), 8.23 (s, 1H), 7.75-7.72 (m, 1H), 7.57-7.54 (m, 4H), 7.43-7.33 (m, 6H), 6.77-6.68 (m, 4H), 6.63-6.58 (m, 2H), 6.09-6.07 (m, 2H). ^{13}C NMR (100 MHz, $CDCl_3$) δ [ppm]: 154.28, 153.98, 144.07, 142.39, 140.75, 140.23, 138.79, 138.65, 133.75, 132.45, 131.57, 129.89, 129.85, 129.20, 128.44, 128.40, 123.33, 121.95, 115.76, 113.60. MS (EI): *m/z* 463 [M^+]. Elemental

analysis (%) for $C_{32}H_{21}N_3O$: C 82.92, H 4.57, N 9.07; Found: C 82.96, H 4.55, N 9.14.

2,3-bis(4-(10*H*-phenoxazin-10-yl)phenyl)-6-(10*H*-phenoxazin-10-yl)-quinoxaline (**DBQ-3PXZ**): it was prepared by the same procedure with **SBDBQ-PXZ** excepting using the key intermediate 3BrDBQ (1.20 g, 2.31 mmol) to replace SBDBQ. **DBQ-3PXZ** is an orange powder (1.85 g, yield: 97%). ^{1}H NMR (400 MHz, $CDCl_3$) δ [ppm]: 8.47 (d, J = 12.0 Hz, 1H), 8.31 (s, 1H), 7.83-7.80 (m, 5H), 7.42 (t, J = 6.0 Hz, 4H), 6.79-6.69 (m, 9H), 6.65-6.63 (m, 6H), 6.54-6.51 (m, 3H), 6.13 (d, J = 8.0 Hz, 2H), 5.96 (t, J = 8.0 Hz, 4H). ^{13}C NMR (100 MHz, $CDCl_3$) δ [ppm]: 153.34, 153.11, 144.16, 143.92, 142.58, 141.02, 140.89, 140.15, 140.11, 138.73, 138.62, 133.88, 133.87, 133.62, 133.11, 132.67, 132.63, 131.60, 131.07, 131.02, 123.45, 123.36, 122.19, 121.68, 115.91, 115.64, 113.66. MS (EI): *m/z* 826 [M$^+$]. Elemental analysis (%) for $C_{56}H_{35}N_5O_3$: C 81.44, H 4.27, N 8.48; Found: C 81.26, H 4.36, N 8.53.

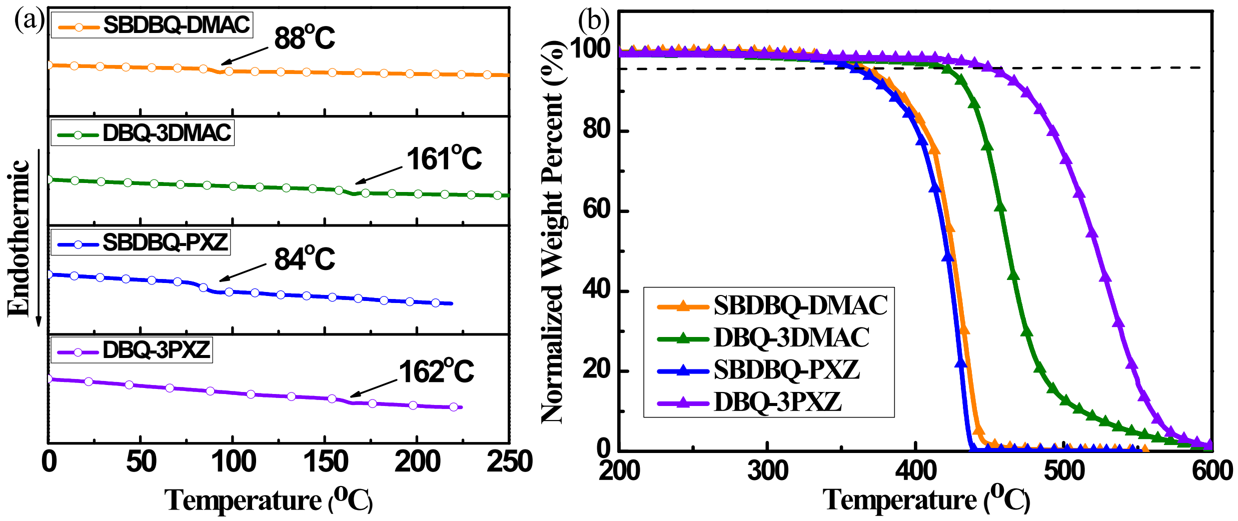

Figure S1. (a) Differential scanning calorimetry (DSC) curves and (b) thermal gravity analysis (TGA) curves of the four target compounds.

Table S1. Values from DFT calculation.

Compound	α[a] [º]	α[b] [º]	HOMO [eV]	LUMO [eV]	S_1 [eV]	T_1 [eV]	ΔE_{ST} [eV]
**SBDBQ-DMAC**	87	-	-4.91	-2.11	2.90	2.66	0.24
**DBQ-3DMAC**	88	88/87	-4.93	-2.35	2.65	2.61	0.04
**SBDBQ-PXZ**	84	-	-4.68	-2.16	2.73	2.66	0.07
**DBQ-3PXZ**	83	75/77	-4.76	-2.45	2.44	2.40	0.04

[a]The dihedral angle between the donor unit on the 6-position of quinoxaline and the quinoxaline plane. [b]The dihedral angles between the two peripheral donor units and the benzene ring plane.

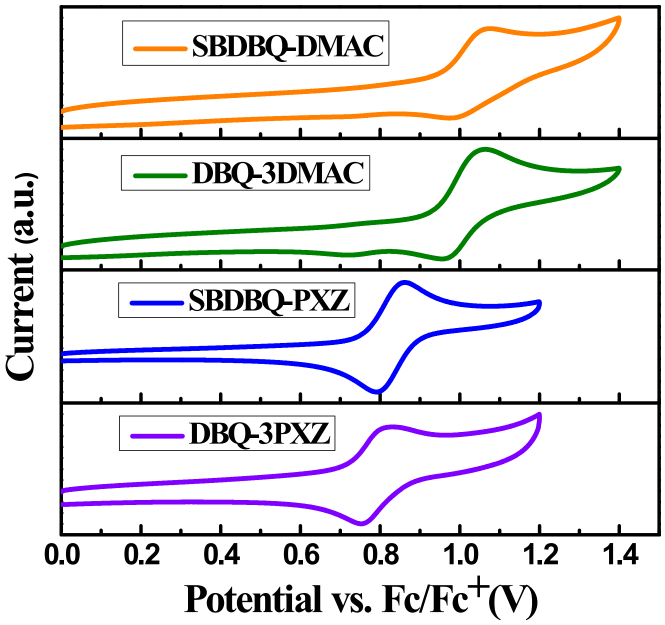

Figure S2. Cyclic voltammogram of target compounds in CH_2Cl_2 for oxidation scan.

Table S2.Thermal, photophysical, and electrochemical data of all compounds.

Compound	T_d[a]/T_g[b] [°C]	λ_{abs}[c] [nm]	$\lambda_{Fl,max}$[d] [nm]	$\lambda_{Ph,max}$[d] [nm]	ΔE_{ST}[e] [eV]	ΔE_g[f] [eV]	HOMO[g]/LUMO[h] [eV]
SBDBQ-DMAC	370/88	345/431	541	555	0.06	2.45	-5.28/-2.83
DBQ-3DMAC	424/161	343/423	551	567	0.06	2.45	-5.26/-2.81
SBDBQ-PXZ	363/84	337/454	594	613	0.07	2.35	-5.17/-2.82
DBQ-3PXZ	455/162	329/436	618	627	0.03	2.27	-5.14/-2.87

[a]Obtained by TGA. [b]Obtained by DSC. [c]Measured in film at room temperature. [d]Measured in film at room temperature and 77 K. [e]$\Delta E_{ST}= E_S- E_T$. [f]Calculated from the absorption edge of the UV/Vis spectrum. [g]Determined from the onset of the oxidation potential. [h]Deduced from HOMO and ΔE_g.

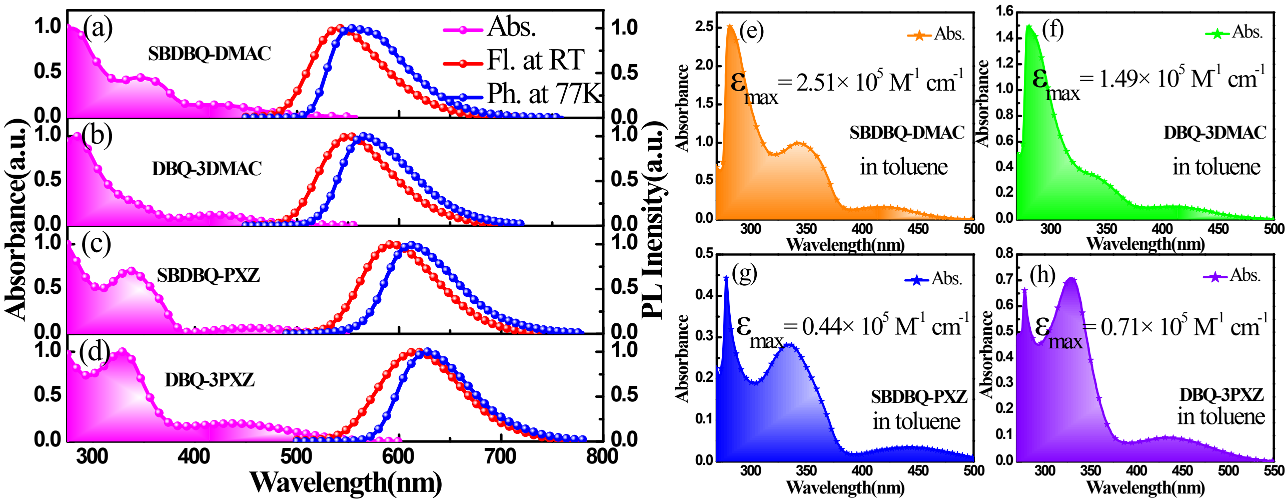

Figure S3. Normalized UV/vis absorption, fluorescence and phosphorescence spectra in film of (a) SBDBQ-DMAC, (b) DBQ-3DMAC, (c) SBDBQ-PXZ, and (d) DBQ-3PXZ, respectively. (e-h) UV/vis absorption spectra and their molar extinction coefficients in toluene (10^{-5} M).

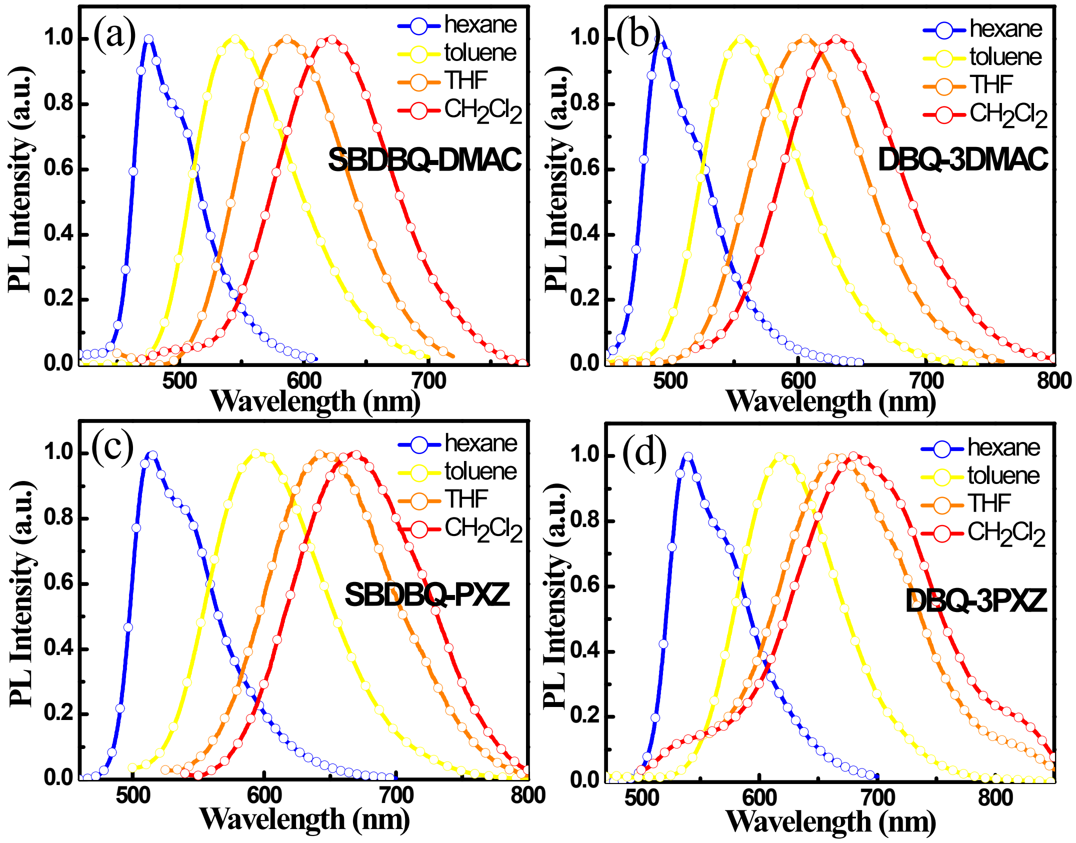

Figure S4. Normalized fluorescence spectra in different solvents of (a) SBDBQ-DMAC, (b) DBQ-3DMAC, (c) SBDBQ-PXZ, and (d) DBQ-3PXZ, respectively.

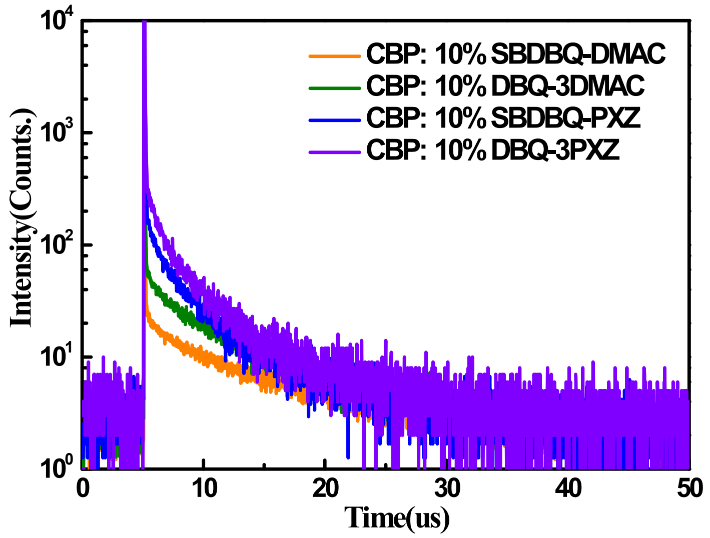

Figure S5. Transient PL decay of CBP:10% TADF in films after the deoxygenation at room temperature.

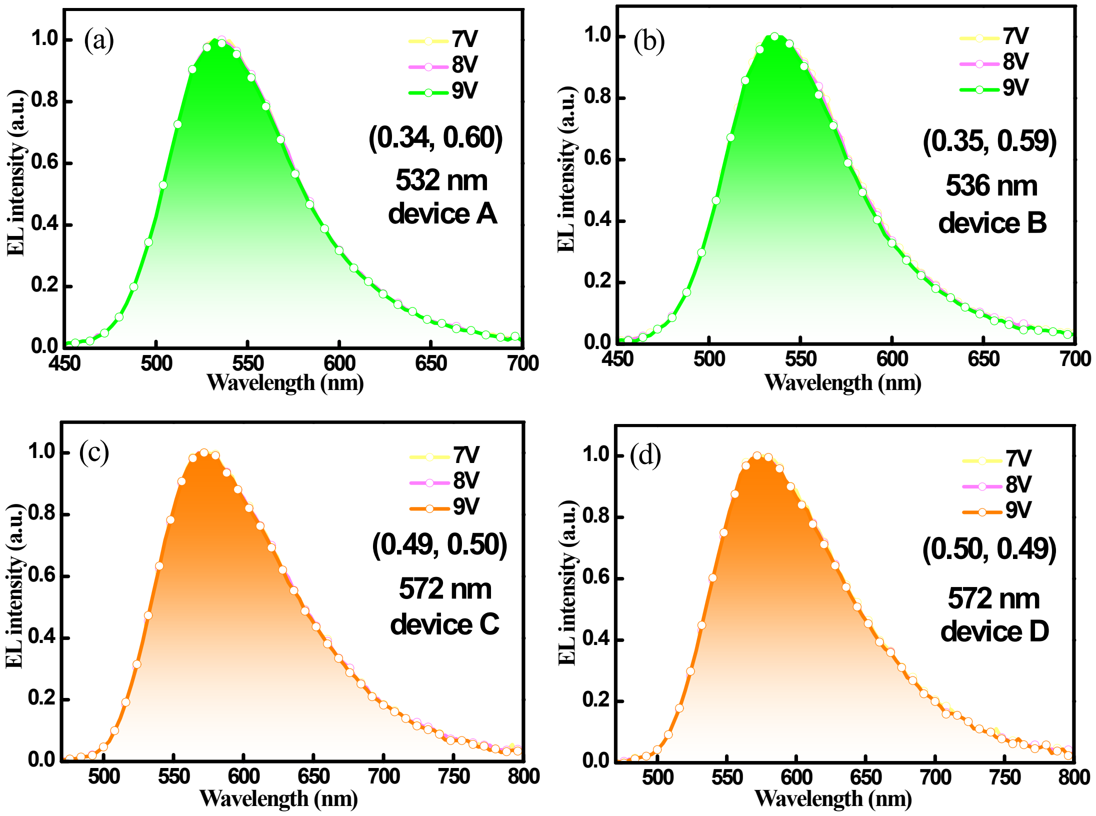

Figure S6. (a-d) EL spectra of the doped devices (A, B, C and D) measured at different voltages, respectively.

Table S3. Comparison of the device data for the representative orange/red OLEDs.

TADF emitter	V_{on}	EQE_{max}	CE_{max}	PE_{max}	CIE	Performance at 100/1000 cd m^{-2}		
	[V]	[%]	[cd A^{-1}]	[lm W^{-1}]	(x, y)	EQE[%]	CE[cd A^{-1}]	PE[lm W^{-1}]
Ac-CNP[S1]	4.7	13.3	38.1	26.1	(0.47,0.51)	12.0/9.1	34.1/26.0	15.3/9.1
Px-CNP[S1]	5.5	3.0	5.8	3.1	(0.53,0.44)	3.0/2.8	5.8/5.1	2.9/1.7
m-Px-2BBP[S2]	2.8	4.2	11.1	~15	(0.58,0.36)	N.A	N.A	N.A
b1[S3]	3.0	12.5	N.A.	N.A.	(0.61,0.39)	8.1/2.3	N.A	N.A
b2[S3]	3.0	9.0	N.A.	N.A.	(0.63,0.37)	5.7/1.7	N.A	N.A
b3[S3]	3.0	9.0	N.A	N.A.	N.A	N.A	N.A	N.A
b4[S3]	3.0	6.9	N.A	N.A	N.A	N.A	N.A	N.A
HAP-3TPA[S4]	4.4	17.5	25.9	22.1	(0.58,0.36)	～10.0/＜5.0	＜20.0/～10.0	＜10.0/＜5.0
4CzTPN-Ph[S5]	N.A.	11.2	N.A.	N.A.	N.A.	N.A	N.A	N.A
**SBDBQ-PXZ**	**3.1**	**11.1**	**29.1**	**23.4**	**(0.49, 0.50)**	**11.0/10.1**	**28.8/26.6**	**20.8/12.9**
**DBQ-3PXZ**	**3.4**	**14.1**	**36.1**	**28.1**	**(0.50,0.49)**	**13.9/11.1**	**35.3/28.4**	**22.9/12.4**

N.A.: not available.

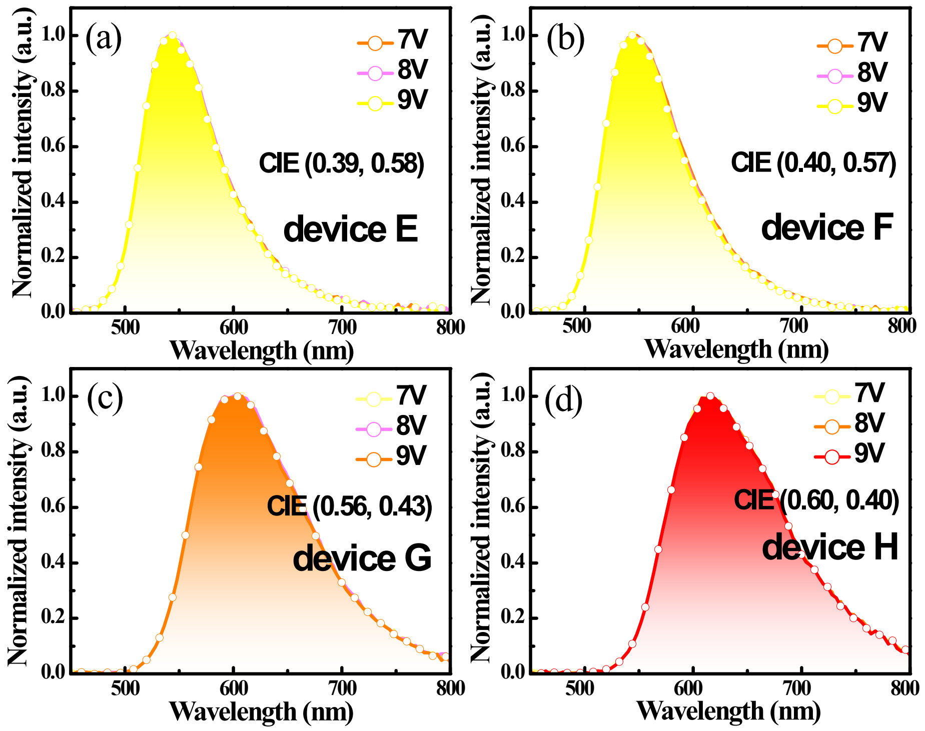

Figure S7. (a-d) EL spectra of the non-doped devices (E, F, G and H) measured at different voltages, respectively.

Table S4. Comparison of the non-doped device performances of the four compounds and representative OLEDs with similar emissions in literatures.

Device	Property	V_{on} [V]	EQE_{max} [%]	CE_{max} [cd A^{-1}]	PE_{max} [lm W^{-1}]	LE_{max} [cd m^{-2}]	Peak [nm]	Reference
T_2BT_2	AIE	4.3	2.88	6.81	4.96	13535	590	Chem. Commun.[S6] 2011, 47, 11273.
V_2BV_2	AIE	3.3	2.53	4.24	4.06	10573	616	
BPA2TPAN	AIE	~3.2	1.4	2.7	1.9	2697	603	J. Mater. Chem. C.[S7] 2014, 2, 7552.
BNA2TPAN	AIE	~3.2	1.3	2.2	1.5	2947	606	
TTB	AIE	3.2	3.5	6.4	6.3	15584	604	Chem. Commun.[S8] 2015, 51, 7321.
TNB	AIE	3.2	3.9	7.5	7.3	16396	604	
**SBDBQ-PXZ**	**AIE+TADF**	**2.4**	**5.6**	**10.5**	**12.0**	**21050**	**608**	**This work**
**DBQ-3PXZ**	**AIE+TADF**	**2.8**	**5.3**	**7.5**	**6.2**	**13167**	**616**	**This work**
$(MesB)_2DMTPS$	AIE	6.9	2.25	7.4	3.2	10500	540	Adv. Funct. Mater.[S9] 2014, 24, 3621.
$(MesB)_2HPS$	AIE	5.4	2.62	8.4	4.1	15200	548	
$(MesB)_2MPPS$	AIE	7.5	2.13	6.6	2.4	9610	552	
DBT-BZ-PXZ	AIE+TADF	2.9	9.2	26.6	27.9	N.A.	557	Chem. Mater.[S10] 2017, 29, 3623.
DBT-BZ-PTZ	AIE+TADF	2.7	9.7	26.5	29.1	N.A.	563	
**SBDBQ-DMAC**	**AIE+TADF**	**2.8**	**10.1**	**35.4**	**32.7**	**14578**	**544**	**This work**
**DBQ-3DMAC**	**AIE+TADF**	**2.6**	**12.0**	**41.2**	**45.4**	**29843**	**548**	**This work**

N.A.: not available.

References

[S1] I. S. Park, S. Y. Lee, C. Adachi and T. Yasuda, *Adv. Funct. Mater.*, 2016, **26**, 1813.

[S2] S. Y. Lee, T. Yasuda, Y. S. Yang, Q. Zhang and C. Adachi, *Angew. Chem. Int. Ed.*, 2014, **53**, 6402.

[S3] Q. Zhang, H. Kuwabara, W. J. Potscavage, Jr., S. Huang, Y. Hatae, T. Shibata and C. Adachi, *J. Am. Chem. Soc.*, 2014, **136**, 18070.

[S4] J. Li, T. Nakagawa, J. MacDonald, Q. Zhang, H. Nomura, H. Miyazaki and C. Adachi, *Adv. Mater.*, 2013, **25**, 3319.

[S5] H. Uoyama, K. Goushi, K. Shizu, H. Nomura and C. Adachi, *Nature*, 2012, **492**, 234.

[S6] H. Li, Z. Chi, X. Zhang, B. Xu, S. Liu, Y. Zhang, J. Xu, *Chem Commun.*, 2011, **47**, 11273.

[S7] Y. Gong, J. Liu, Y. Zhang, G. He, Y. Lu, W. B. Fan, W. Z. Yuan, J. Z. Sun, Y. Zhang, *J. Mater. Chem. C.*, 2014, **2**, 7552.

[S8] W. Qin, J. W. Lam, Z. Yang, S. Chen, G. Liang, W. Zhao, H. S. Kwok, B. Z. Tang, *Chem Commun.*, 2015, **51**, 7321.

[S9] L. Chen, Y. Jiang, H. Nie, P. Lu, H. H. Y. Sung, I. D. Williams, H. S. Kwok, F. Huang, A. Qin, Z. Zhao, B. Z. Tang, *Adv. Funct. Mater.*, 2014, **24**, 3621.

[S10] J. Guo, X.-L. Li, H. Nie, W. Luo, R. Hu, A. Qin, Z. Zhao, S.-J. Su, B. Z. Tang, *Chem. Mater.*, 2017, **29**, 3623.

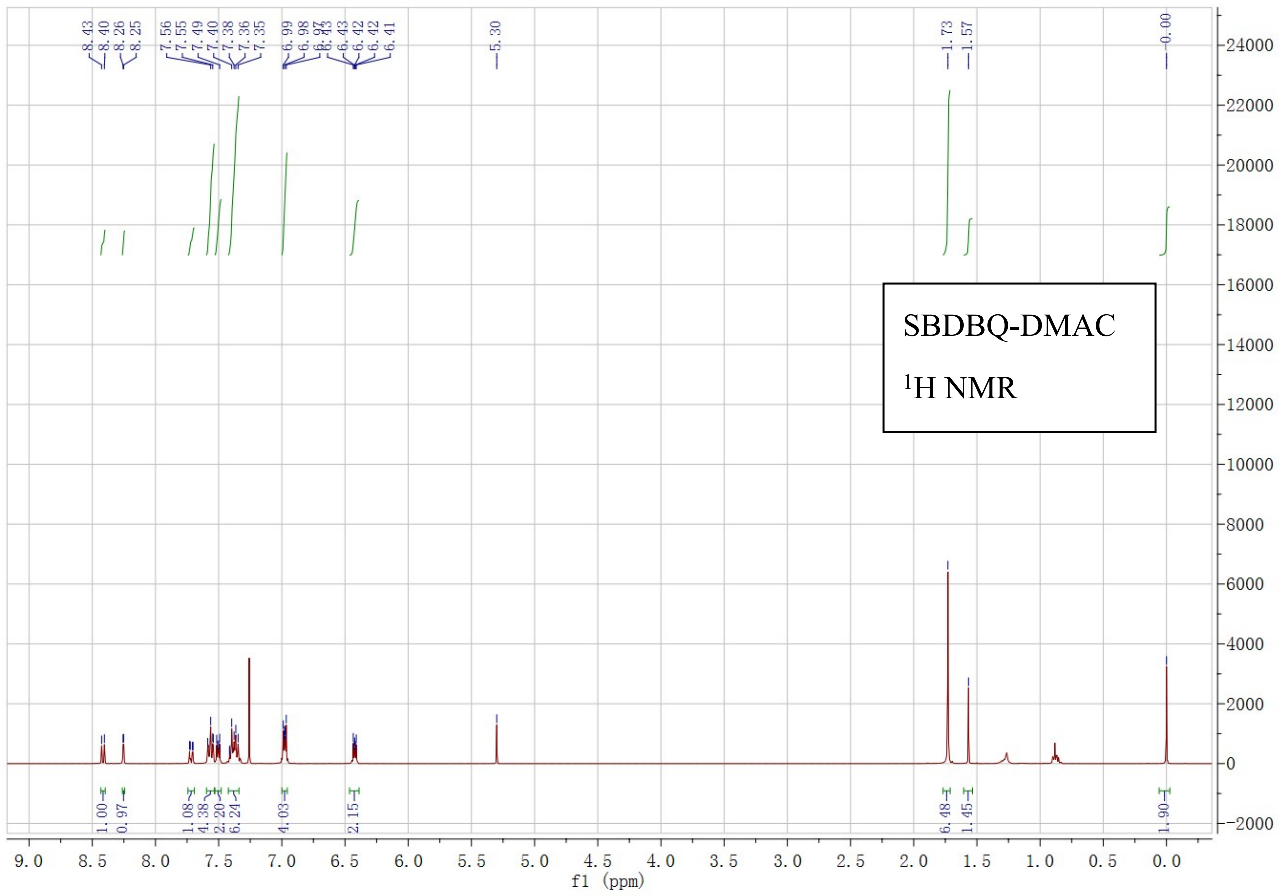
SBDBQ-DMAC
^{1}H NMR
f1 (ppm)

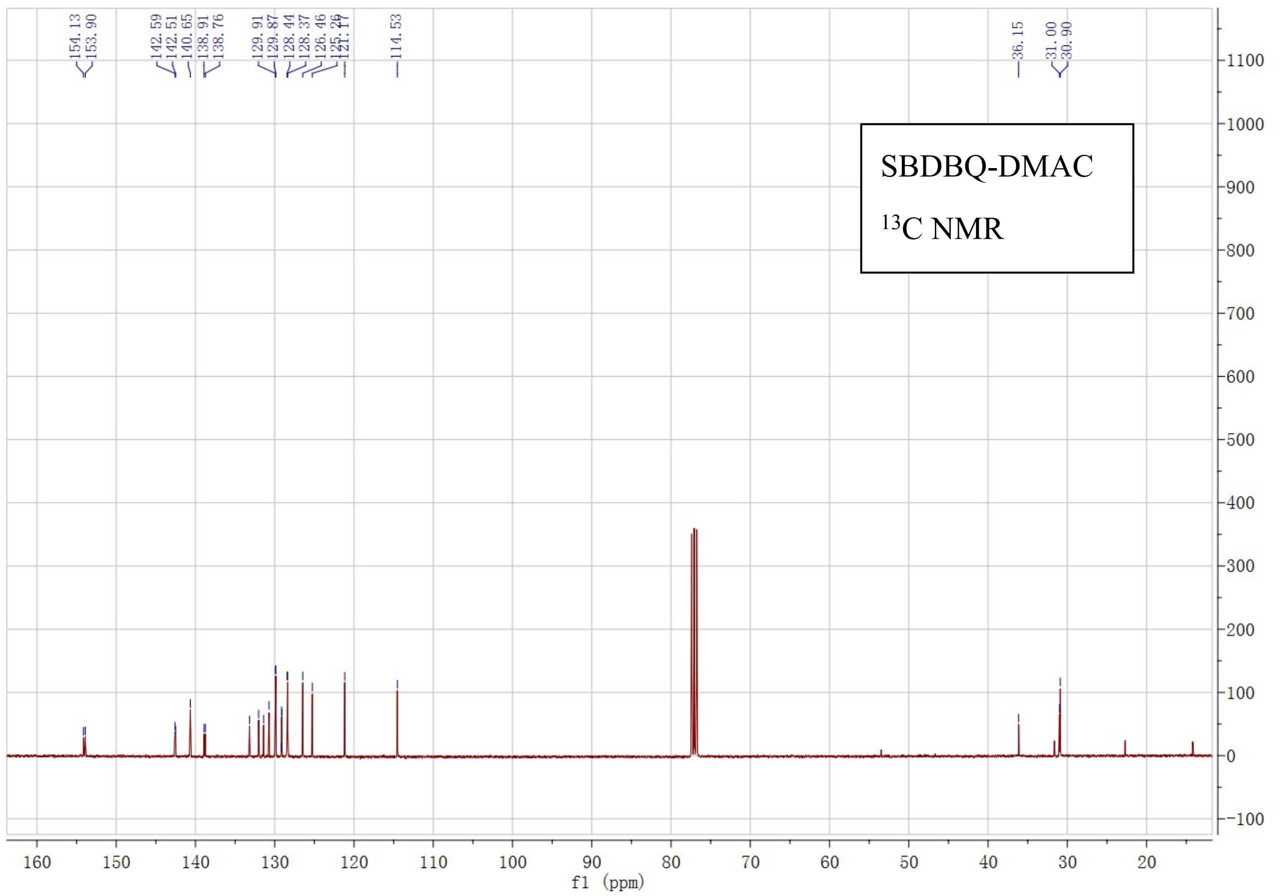
SBDBQ-DMAC
^{13}C NMR
f1 (ppm)

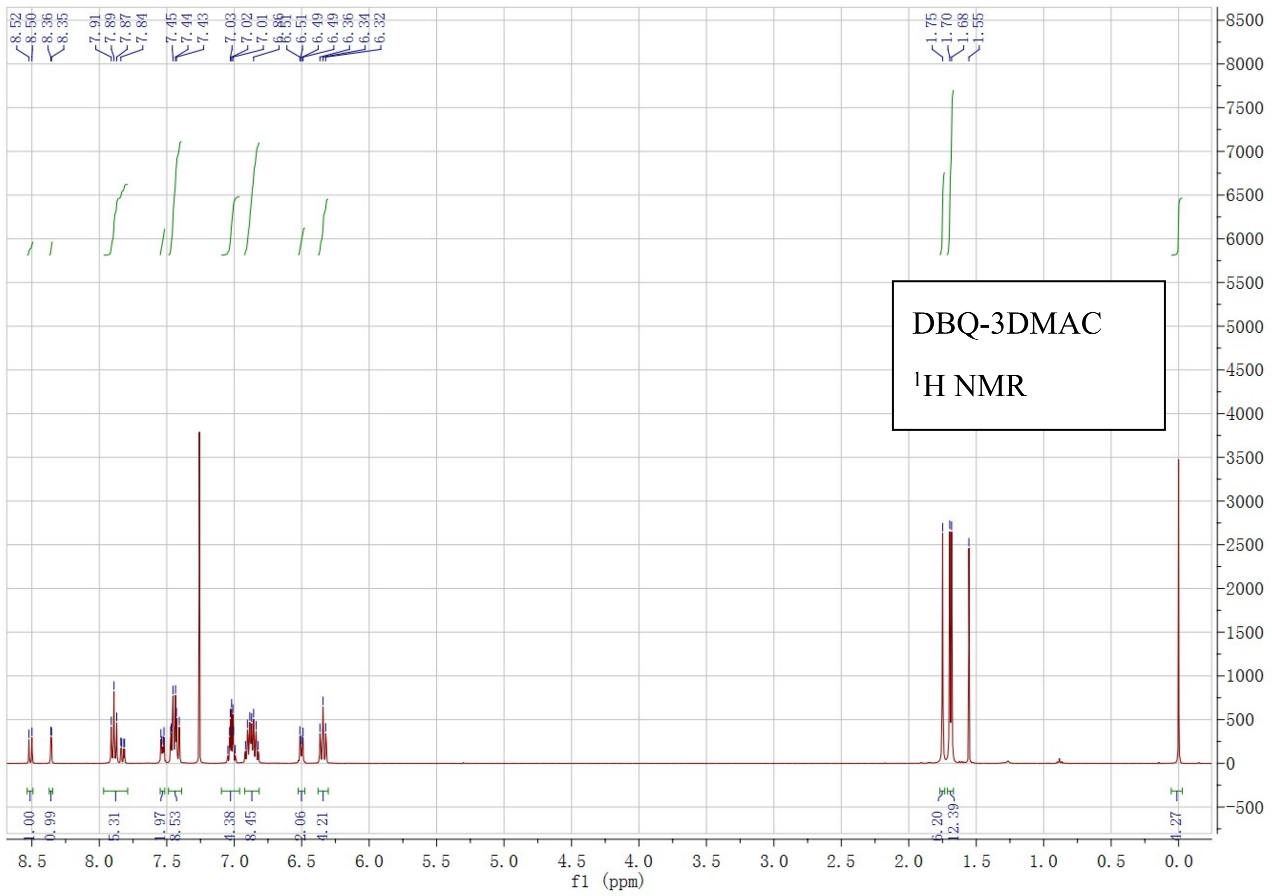
DBQ-3DMAC
1H NMR
f1 (ppm)

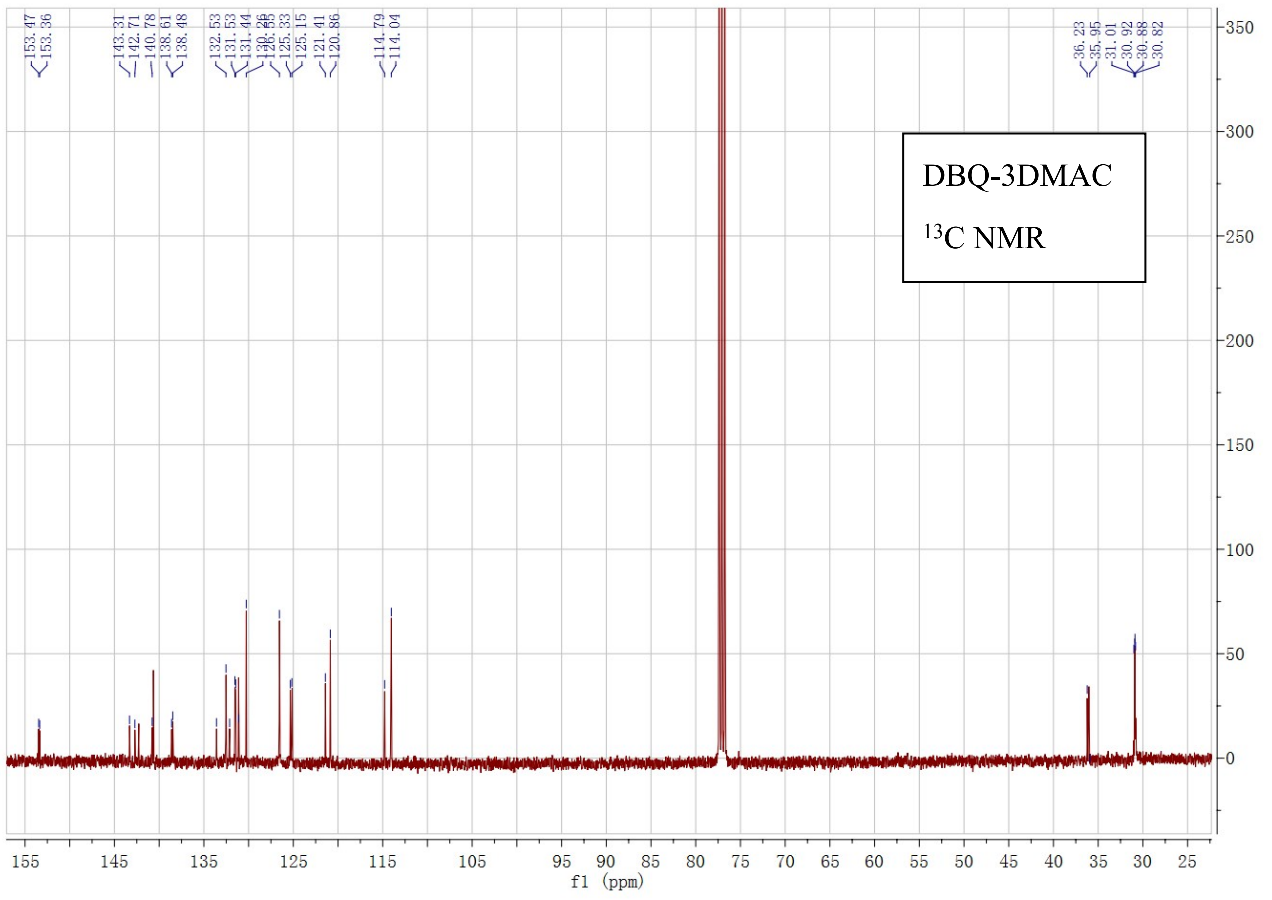
DBQ-3DMAC
13C NMR
f1 (ppm)

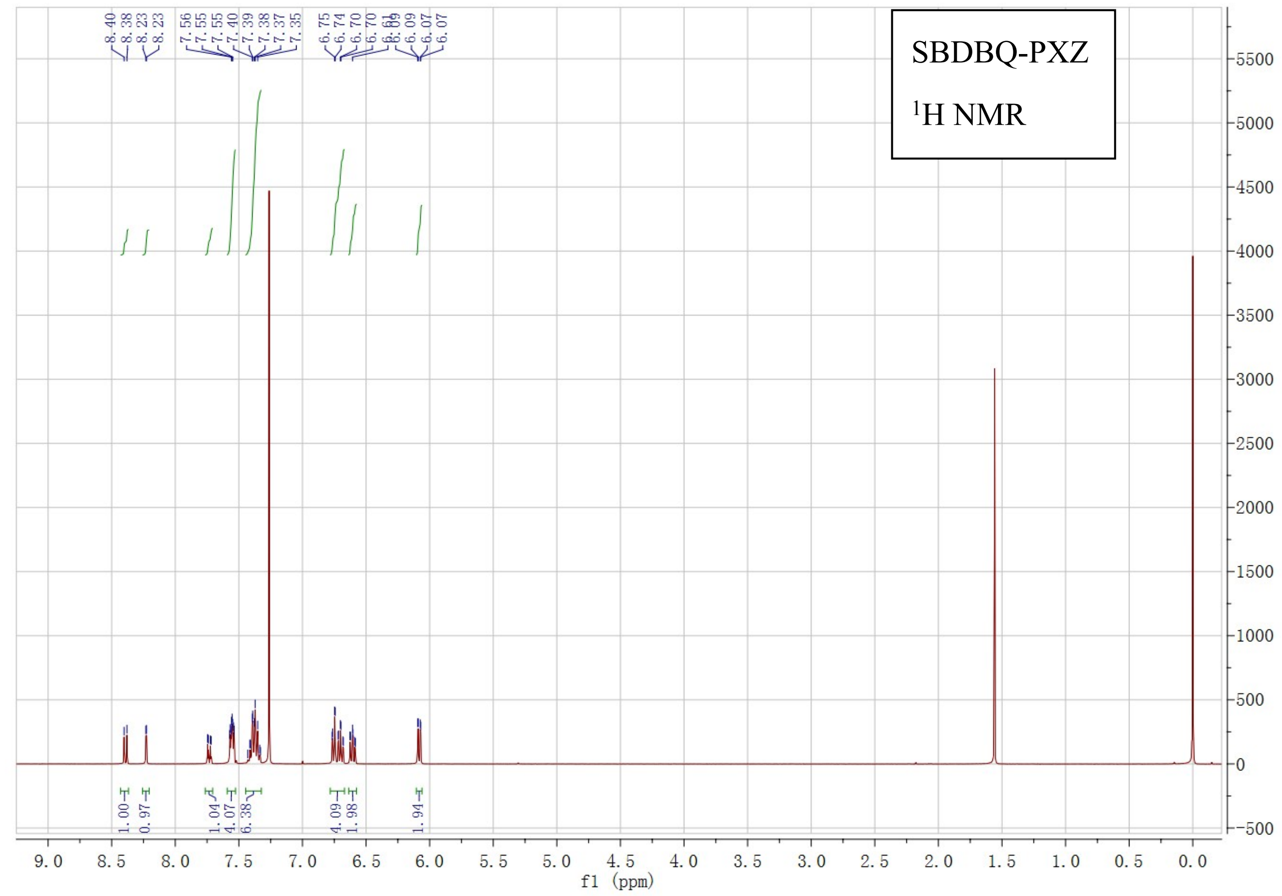
SBDBQ-PXZ
1H NMR
f1 (ppm)

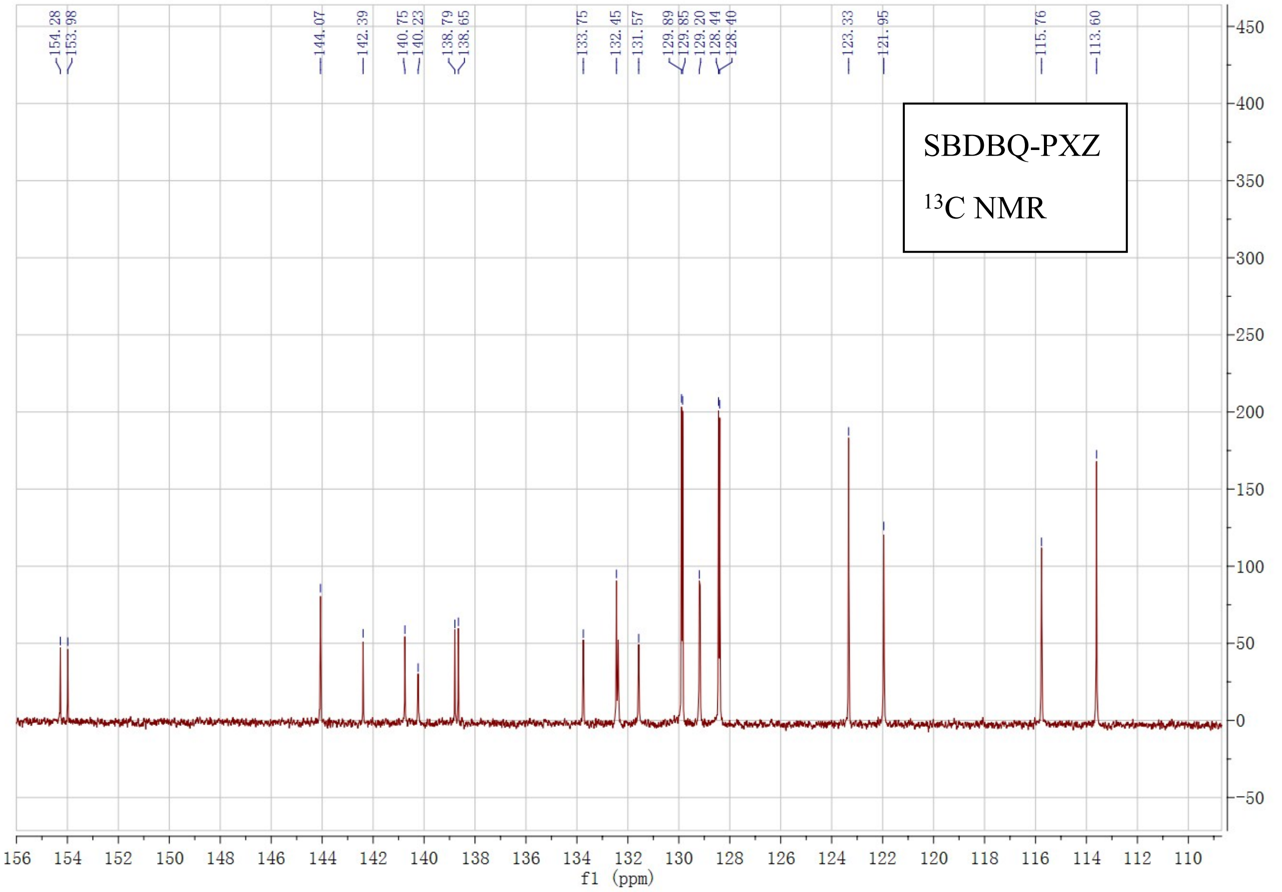
SBDBQ-PXZ
13C NMR
f1 (ppm)

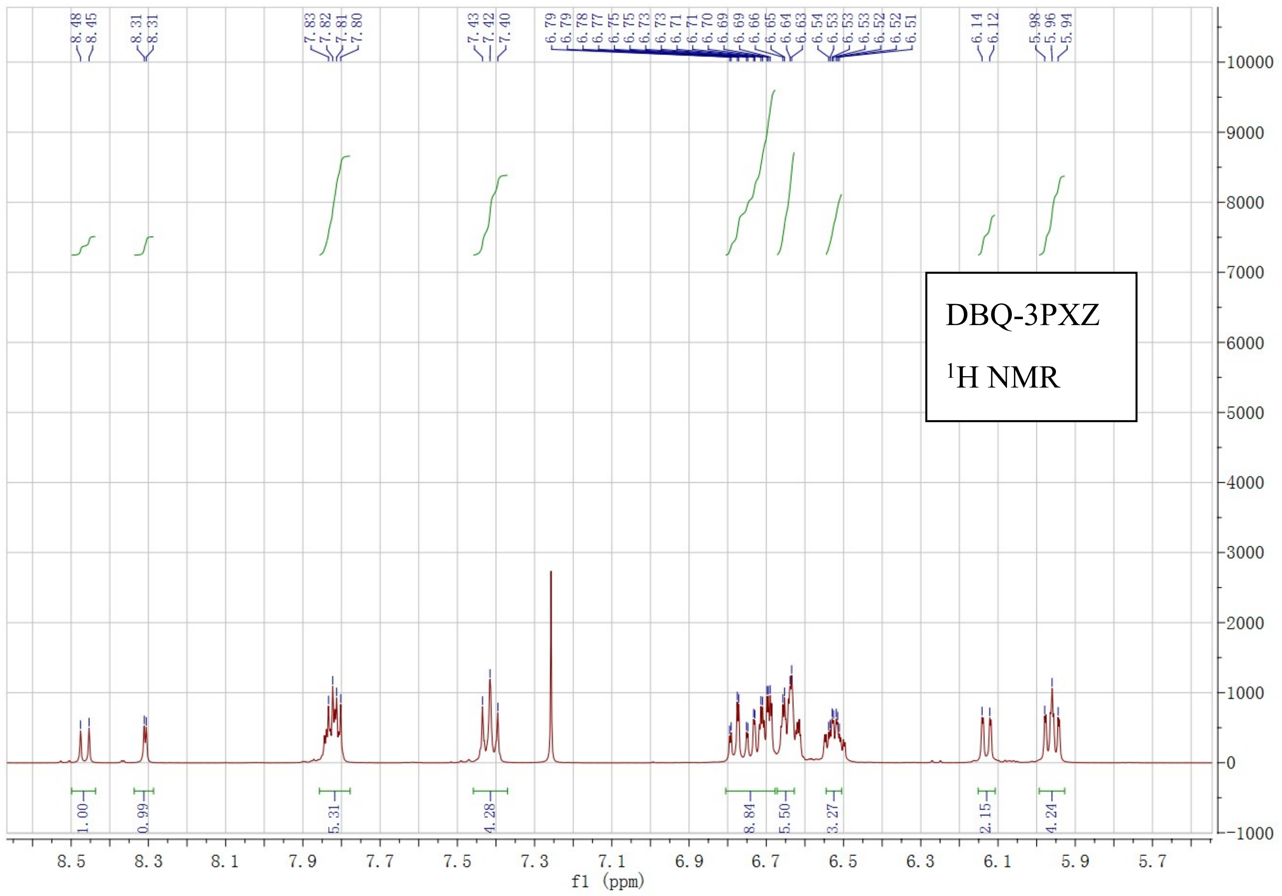
DBQ-3PXZ
^{1}H NMR
f1 (ppm)

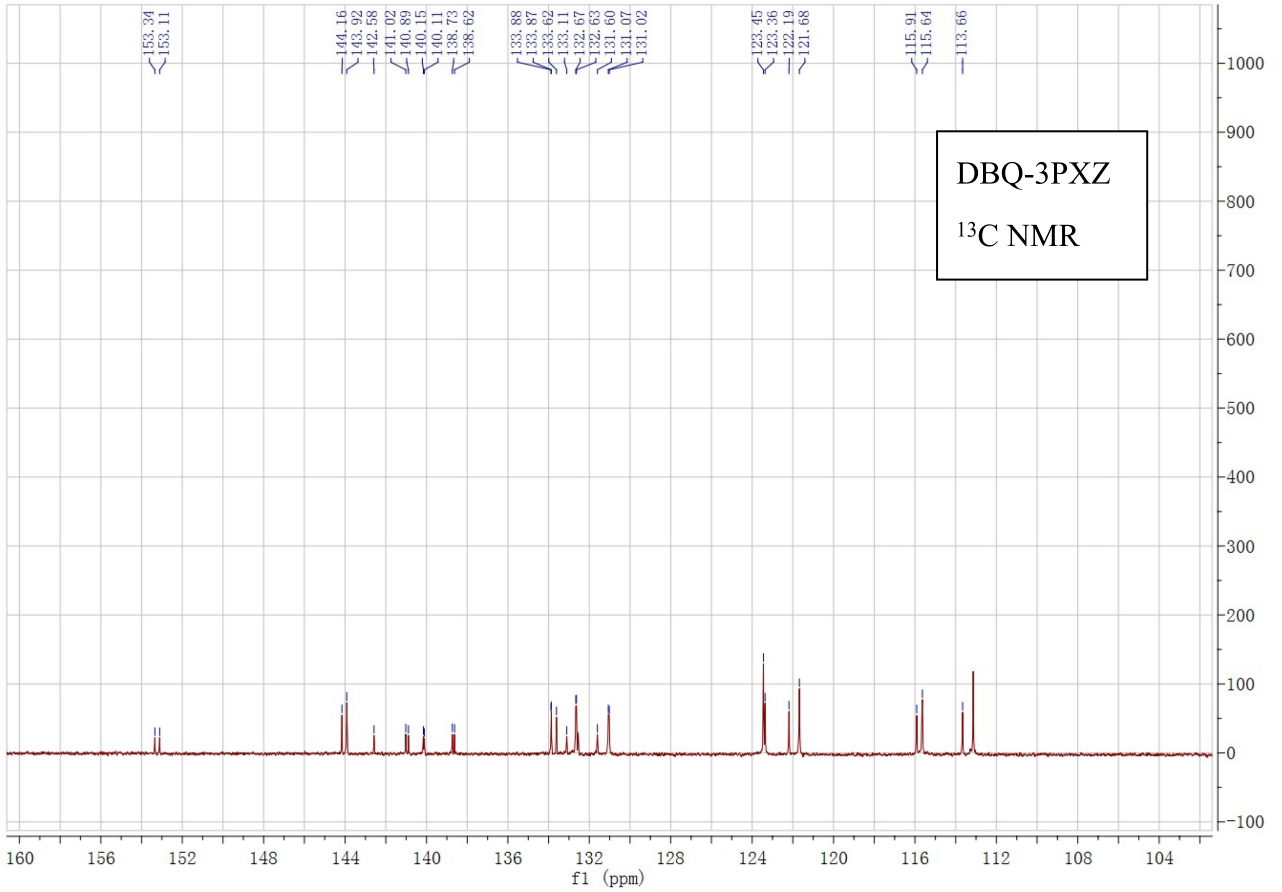
DBQ-3PXZ
^{13}C NMR
f1 (ppm)

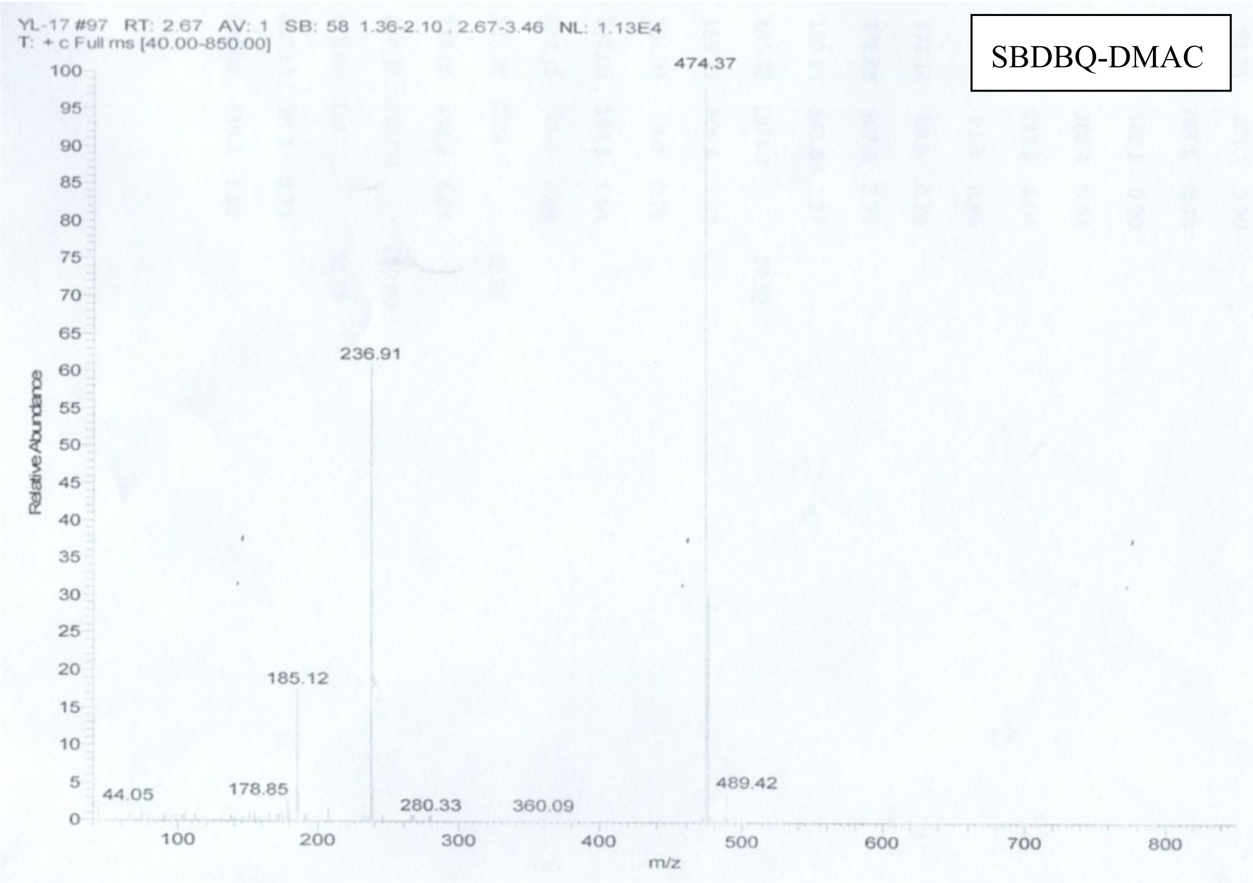
YL-17 #97 RT: 2.67 AV: 1 SB: 58 1.36-2.10 , 2.67-3.46 NL: 1.13E4
T: + c Full ms [40.00-850.00]
SBDBQ-DMAC
474.37
236.91
185.12
44.05
178.85
280.33
360.09
489.42
Relative Abundance
m/z

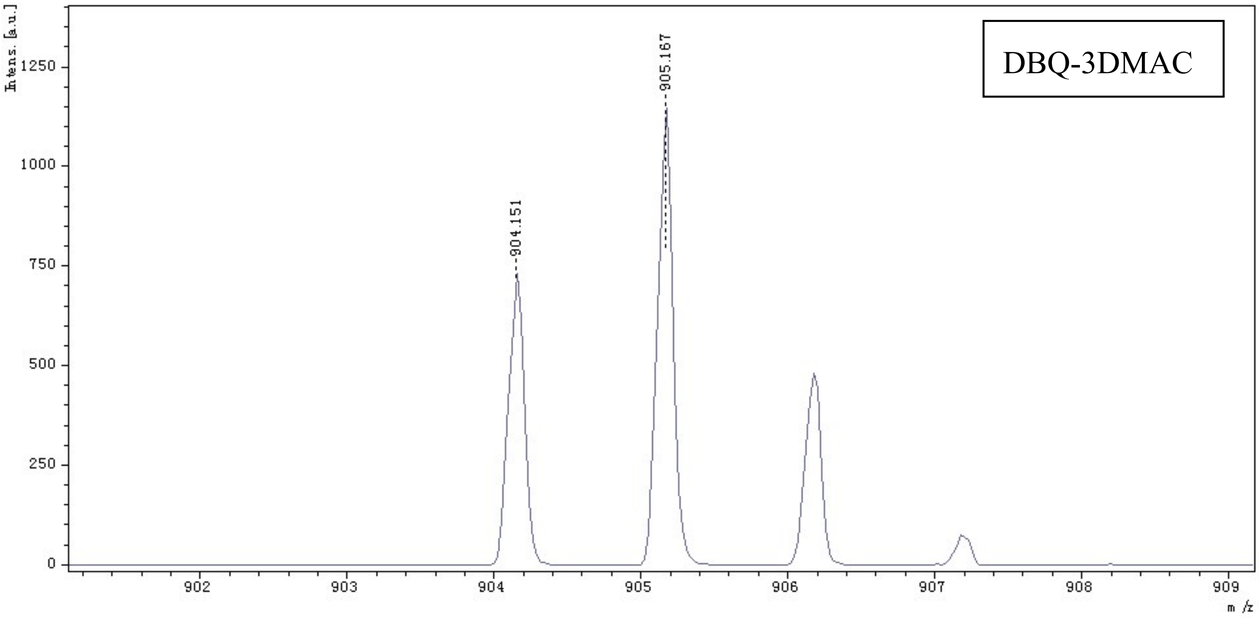
DBQ-3DMAC
905.167
904.151
Intens. [a.u.]
m/z

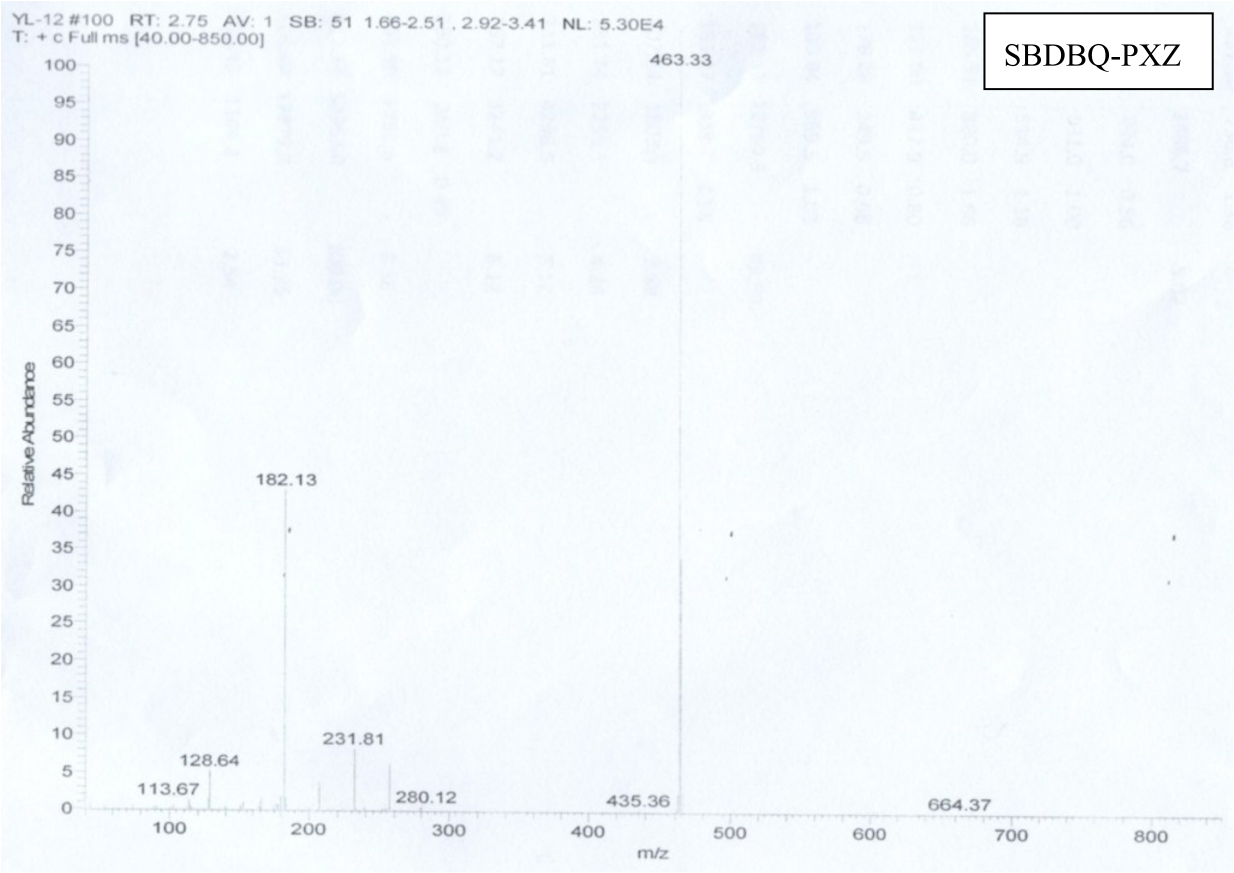
YL-12 #100 RT: 2.75 AV: 1 SB: 51 1.66-2.51 , 2.92-3.41 NL: 5.30E4
T: + c Full ms [40.00-850.00]
SBDBQ-PXZ
463.33
182.13
231.81
128.64
113.67
280.12
435.36
664.37
Relative Abundance
m/z

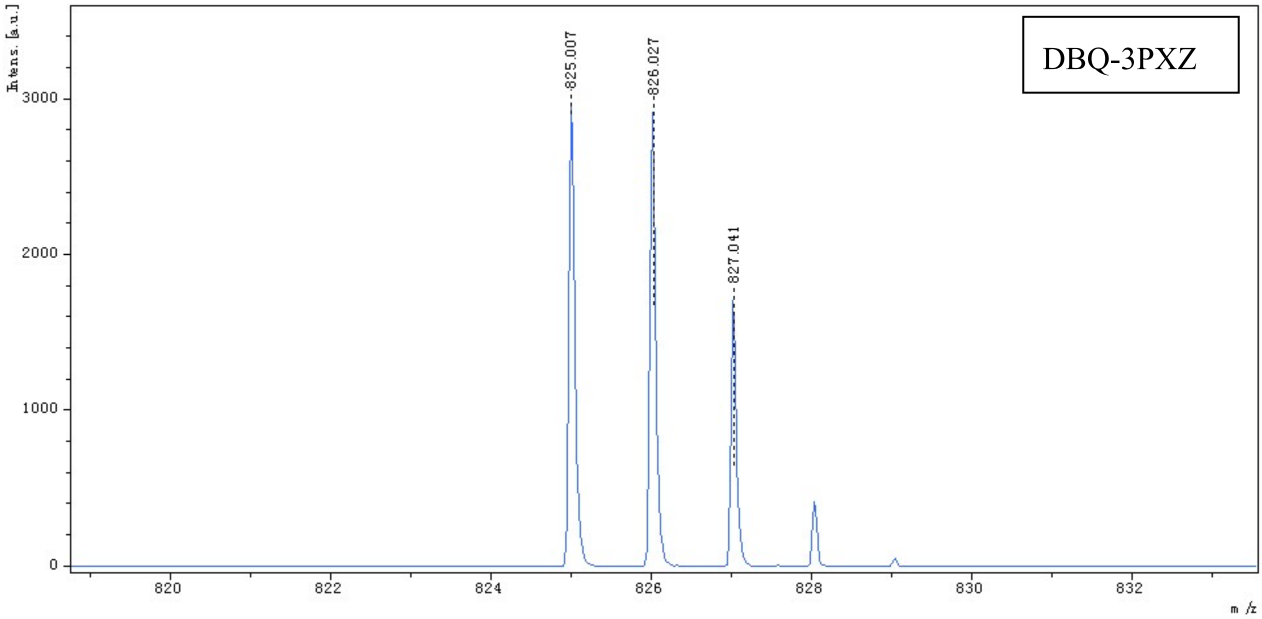
DBQ-3PXZ
Intens. [a.u.]
825.007
826.027
827.041
m/z
